# High‐efficiency production of bisabolene from waste cooking oil by metabolically engineered *Yarrowia lipolytica*

**DOI:** 10.1111/1751-7915.13768

**Published:** 2021-02-19

**Authors:** Yakun Zhao, Kun Zhu, Jian Li, Yu Zhao, Shenglong Li, Cuiying Zhang, Dongguang Xiao, Aiqun Yu

**Affiliations:** ^1^ State Key Laboratory of Food Nutrition and Safety Key Laboratory of Industrial Fermentation Microbiology of the Ministry of Education Tianjin Key Laboratory of Industrial Microbiology College of Biotechnology Tianjin University of Science and Technology No. 29 the 13th Street TEDA Tianjin 300457 China

## Abstract

The natural plant product bisabolene serves as a precursor for the production of a wide range of industrially relevant chemicals. However, the low abundance of bisabolene in plants renders its isolation from plant sources non‐economically viable. Therefore, creation of microbial cell factories for bisabolene production supported by synthetic biology and metabolic engineering strategies presents a more competitive and environmentally sustainable method for industrial production of bisabolene. In this proof‐of‐principle study, for the first time, we engineered the oleaginous yeast *Yarrowia lipolytica* to produce α‐bisabolene, β‐bisabolene and γ‐bisabolene through heterologous expression of the α‐bisabolene synthase from *Abies grandis*, the β‐bisabolene synthase gene from *Zingiber officinale* and the γ‐bisabolene synthase gene from *Helianthus annuus* respectively. Subsequently, two metabolic engineering approaches, including overexpression of the endogenous mevalonate pathway genes and introduction of heterologous multidrug efflux transporters, were employed in order to improve bisabolene production. Furthermore, the fermentation conditions were optimized to maximize bisabolene production by the engineered *Y. lipolytica* strains from glucose. Finally, we explored the potential of the engineered *Y. lipolytica* strains for bisabolene production from the waste cooking oil. To our knowledge, this is the first report of bisabolene production in *Y. lipolytica* using metabolic engineering strategies. These findings provide valuable insights into the engineering of *Y. lipolytica* for a higher‐level production of bisabolene and its utilization in converting waste cooking oil into various industrially valuable products.

## Graphical abstract

To our knowledge, we have unprecedentedly verified the function of α‐bisabolene synthase, β‐bisabolene synthase and γ‐bisabolene synthase in *Y. lipolytica* and successfully employed the enzyme to synthesize bisabolene in *Y. lipolytica* for the first time. Our study reports for the first time the utilization of terpenoid efflux pumps to improve the secretion, and thus the production, of bisabolene in microbial cell factories. We demonstrated for the first time the conversion of waste cooking oil to bisabolene, which has important potential applications in waste management as well as economical and sustainable production of valuable bisabolene from waste feedstocks.

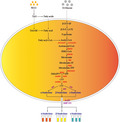

## Introduction

Bisabolene (C_15_H_24_) is the simplest monocyclic sesquiterpene and also a bioactive compound that commonly exists in natural plant essential oils. It has three structural isomers, namely, α‐bisabolene, β‐bisabolene and γ‐bisabolene, and each isomer has distinctly different properties and applications. Currently, the plant terpenoid bisabolene has a wide range of applications in cosmetic, chemical, pharmaceutical and nutraceutical industries (Peralta‐Yahya *et al*., 2011; Davies *et al*., 2014). Traditionally, bisabolene is extensively used as a high‐value fragrance and flavour compound in many industries because it has a highly pleasant fruity and balsamic aroma (Wang *et al*., 2017). For example, β‐bisabolene has an odour similar to sesame oil and thus can be used as a food flavouring. Furthermore, bisabolene is being investigated as an anti‐inflammatory and anti‐cancer agent and thus would be of great benefit to the medical community (Jou *et al*., 2016). In addition, bisabolene could also serve as an essential starting material for the synthesis of various commercially valuable products, such as a novel biosynthetic alternative to the D2 diesel. (Peralta‐Yahya *et al*., 2011; Davies *et al*., 2014).

At present, the industrial production of bisabolene is mostly achieved by direct extraction from plant tissues. However, this method has many disadvantages, such as limited raw material source, low yield of the product and complicated separation steps (Lopresto *et al*., 2014). Likewise, chemical syntheses of bisabolene suffer from the complexity of the production equipment and low conversion rate of raw materials (Liu and Khosla, 2010). These processes are also energy‐intensive and can cause environmental issues. As a result, there is an ever‐increasing demand to develop alternative and renewable routes to bisabolene. Among the alternative approaches, biosynthesizing bisabolene in microbial cell factories generated by metabolic engineering and synthetic biology is becoming a highly promising strategy that can overcome the aforementioned bottleneck, making bisabolene production more sustainable and environmentally friendly.

The industrial microbe *Yarrowia lipolytica* is an unconventional oleaginous yeast, which was classified by the US Food and Drug Administration as generally regarded as safe (GRAS) (Madzak and Beckerich, 2013). One of the distinguishing metabolic features of *Y. lipolytica* is that it is capable of efficiently utilizing a variety of low‐cost hydrophobic substrates for growth (Abghari and Chen, 2014). In recent years, *Y. lipolytica* has demonstrated its versatility and importance as a production host platform by its successful application for a wide range of purposes in metabolic engineering and synthetic biology (Madzak *et al*., 2004; Beopoulos et al., 2009; Abdel‐Mawgoud *et al*., 2018). *Y. lipolytica* has been particularly considered as an attractive host platform for the production of terpenes because its endogenous cytosolic mevalonate (MVA) pathway can give rise to geranyl diphosphate (GPP), farnesyl diphosphate (FPP) and geranylgeranyl diphosphate (GGPP), which are the direct substrates for the biosynthesis of monoterpenes, sesquiterpenes and diterpenes respectively. To date, several plant terpenes have been successfully produced in this engineered yeast. These mainly include the monoterpenes limonene (Cao *et al*., 2016; Pang *et al*., 2019) and linalool (Cao *et al*., 2017), the sesquiterpene farnesene (Liu *et al*., 2019), the triterpene betulinic acid (Jin *et al*., 2019), the tetraterpenes β‐carotene (Gao *et al*., 2017; Larroude *et al*., 2017) and lycopene (Matthäus *et al*., 2014; Schwartz *et al*., 2017).

In this study, we report the engineering of *Y. lipolytica* for the overproduction of the sesquiterpene bisabolene. First, we engineered *Y. lipolytica* to heterologously express the selected genes of α‐bisabolene synthase, β‐bisabolene synthase and γ‐bisabolene synthase to produce the corresponding bisabolene from FPP. To our knowledge, this is the first report of bisabolene production in *Y. lipolytica*. Second, the influence of overexpressing genes involved in the MVA pathway on bisabolene production was examined. Third, we demonstrated that expression of heterologous efflux pumps could lead to increased bisabolene production. Finally, the potential of using waste cooking oil as the carbon source for bisabolene production was investigated with the engineered *Y. lipolytica* strains. The outcome of this work shows that our engineered *Y. lipolytica* can serve as a platform strain for future metabolic engineering efforts to biosynthesize valuable bisabolene‐derived chemicals.

## Results

### Production of bisabolene in Y. lipolytica by introduction of plant bisabolene synthases

**Fig. 1:**
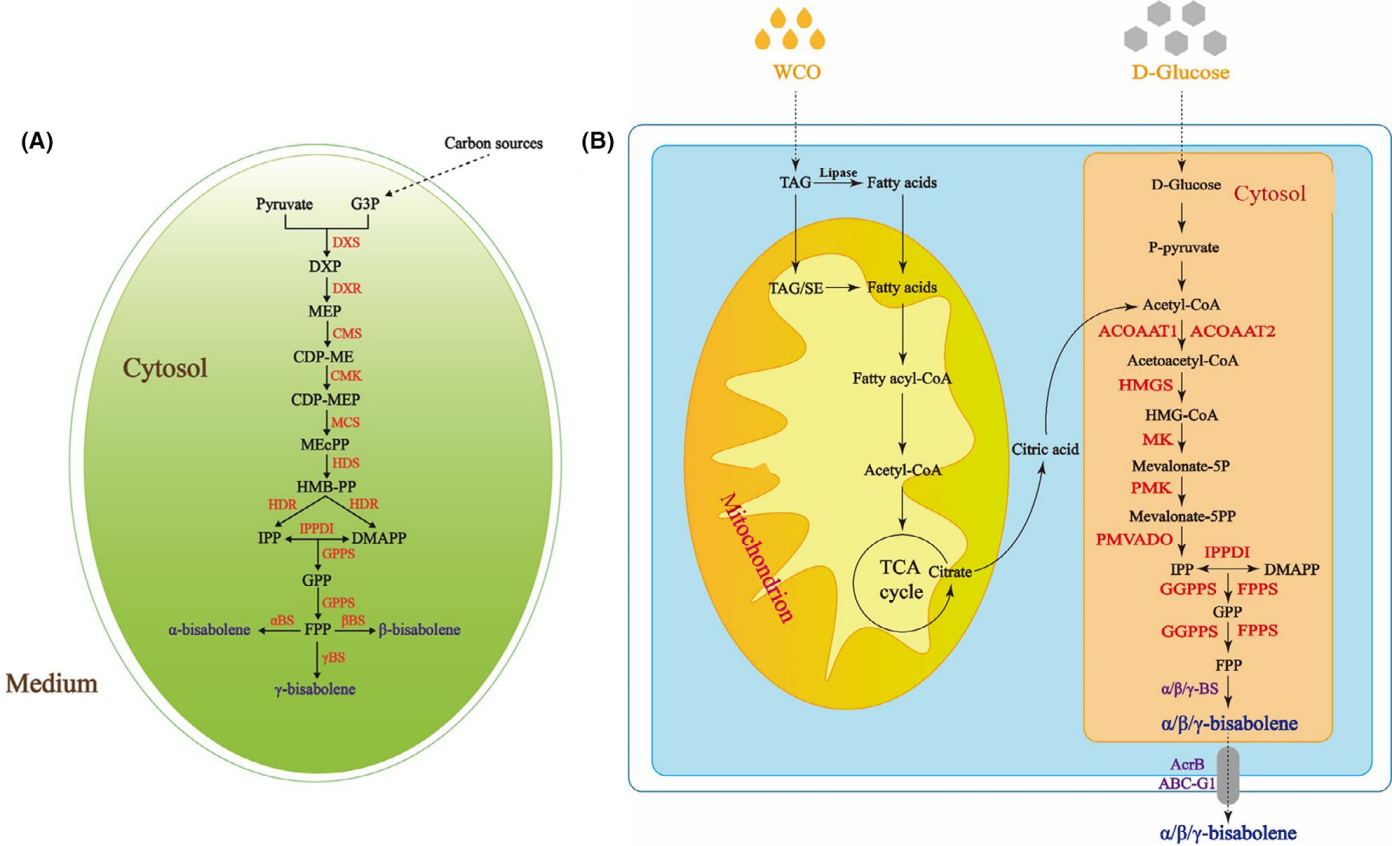
Biosynthesis pathway for bisabolene production in the yeast *Y. lipolytica* and biosynthesis pathway for bisabolene production in the plants. A. Biosynthesis pathway for bisabolene production in plants. Plants produce FPP via the methylerythritol phosphate pathway from pyruvate and glyceraldehyde‐3‐phosphate. The endogenous MEP pathway enzymes are shown in red. DXS: DXP synthase, DXR: DXP‐reductoisomerase, CMS: MEP cytidylyltransferase, CMK: CDP‐ME kinase, MCS: MECDP synthase, HDS: (E)‐4‐hydroxy‐3‐methylbut‐2‐enyl‐diphosphate synthase, HDR: HMBPP reductase, IPPDI: isopentenyl‐diphosphate delta‐isomerase, GPPS: geranyl diphosphate synthase, αBS: *α*‐bisabolene synthase, βBS: β‐bisabolene synthase, γBS: γ‐bisabolene synthase. B. Biosynthesis pathway for bisabolene production in the yeast *Y. lipolytica*. Yeast rely on the MVA pathway to produce FPP from acetyl‐CoA. Since the bisabolene synthase (BS) is not present in *Y. lipolytica*, to construct a complete bisabolene pathway in *Y. lipolytica*, three heterologous genes encoding α‐bisabolene synthase (αBS, from *A. grandis*), β‐bisabolene synthase gene (βBS, from *Z. officinale*) and the γ‐bisabolene synthase gene (γBS, from *H. annuus*) were introduced. The endogenous MVA pathway enzymes and the heterologous enzymes that were overexpressed in the engineered *Y. lipolytica* strains are shown in red and purple respectively. αBS: *α*‐bisabolene synthase, βBS: β‐bisabolene synthase, γBS: γ‐bisabolene synthase ACOAAT1: acetyl‐CoA C‐acetyltransferase 1, ACOAAT2: acetyl‐CoA C‐acetyltransferase 2, HMGS: hydroxymethylglutaryl‐CoA synthase, HMGR: hydroxymethylglutaryl‐CoA reductase, MK: mevalonate kinase, PMK: phosphomevalonate kinase, PMVADO: diphosphomevalonate decarboxylase, IPPDI: isopentenyl‐diphosphate delta‐isomerase, GGPPS: geranylgeranyl diphosphate synthase, type III, FPPS: farnesyl diphosphate synthase.

**Fig. 2:**
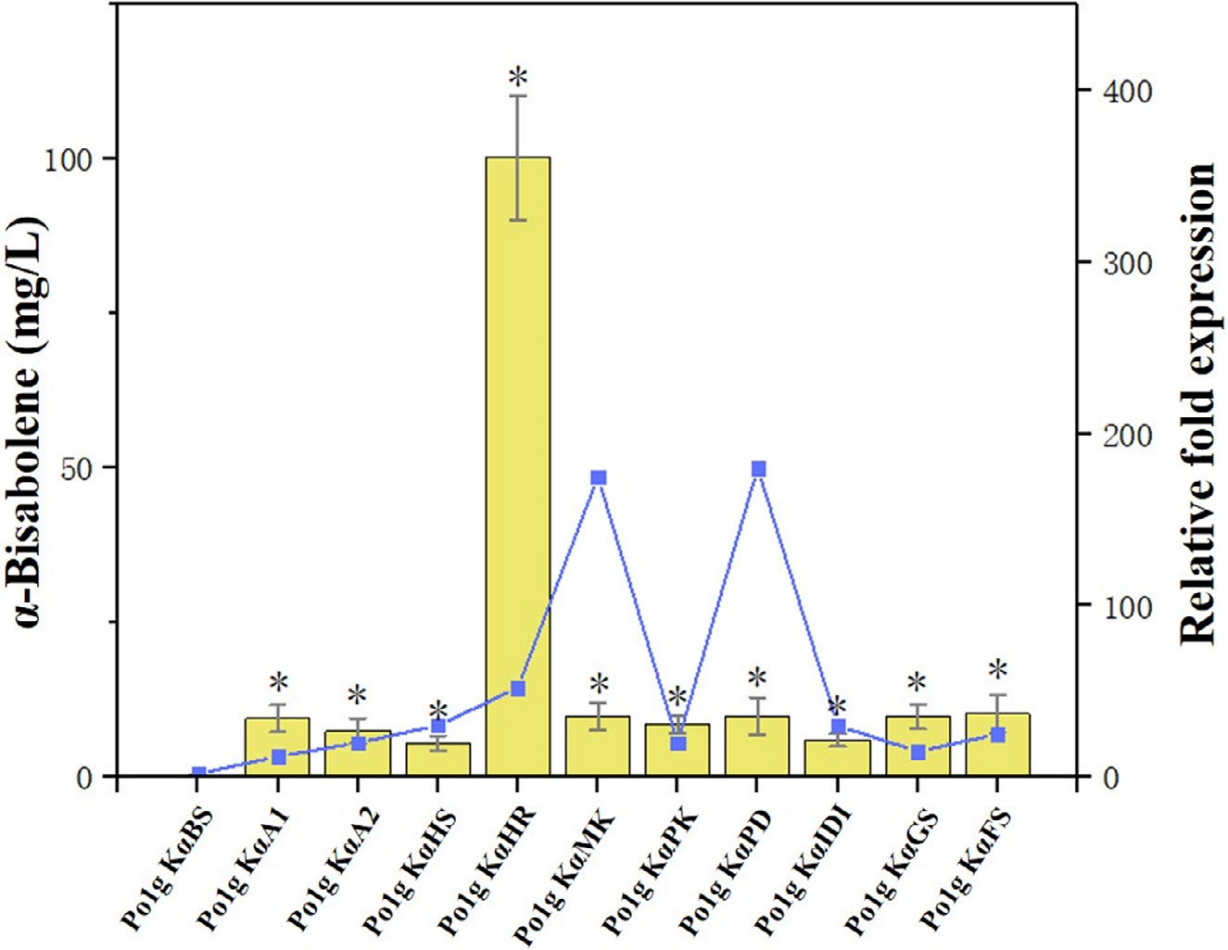
Effects of single‐gene overexpression of genes involved in the MVA pathway on α‐bisabolene production. Ten genes including *ACOAAT1*, *ACOAAT2*, *HMGS*, *HMGR*, *MK*, *PMK*, *PMVADO*, *IPPDI*, *GGPPS* and *FPPS* involved in MVA pathway were overexpressed individually with α‐bisabolene synthase. Bars represent α‐bisabolene titers, and lines represent gene expression increase over controls. Titers of α‐bisabolene were quantified after 5 days of cultivation in shake flasks with 50 ml of liquid YPD medium. The mRNA levels of the ten genes were measured by the SYBR Green I fluorescence method after 3 days of cultivation in shake flasks with 50 ml of liquid YPD medium. Glucose was used as the carbon source. The Po1g KαBS strain cultivated in parallel was used as control, and the gene encoding the *β*‐actin protein was used as the internal standard. Relative gene expression was calculated relative to *β*‐actin by 2^‐▵▵^ *^C^* ^T^ and normalized to controls. All values presented are the mean of three biological replicates ± standard deviation. *P < 0.05, significantly different from control by ANOVA.

**Fig. 3:**
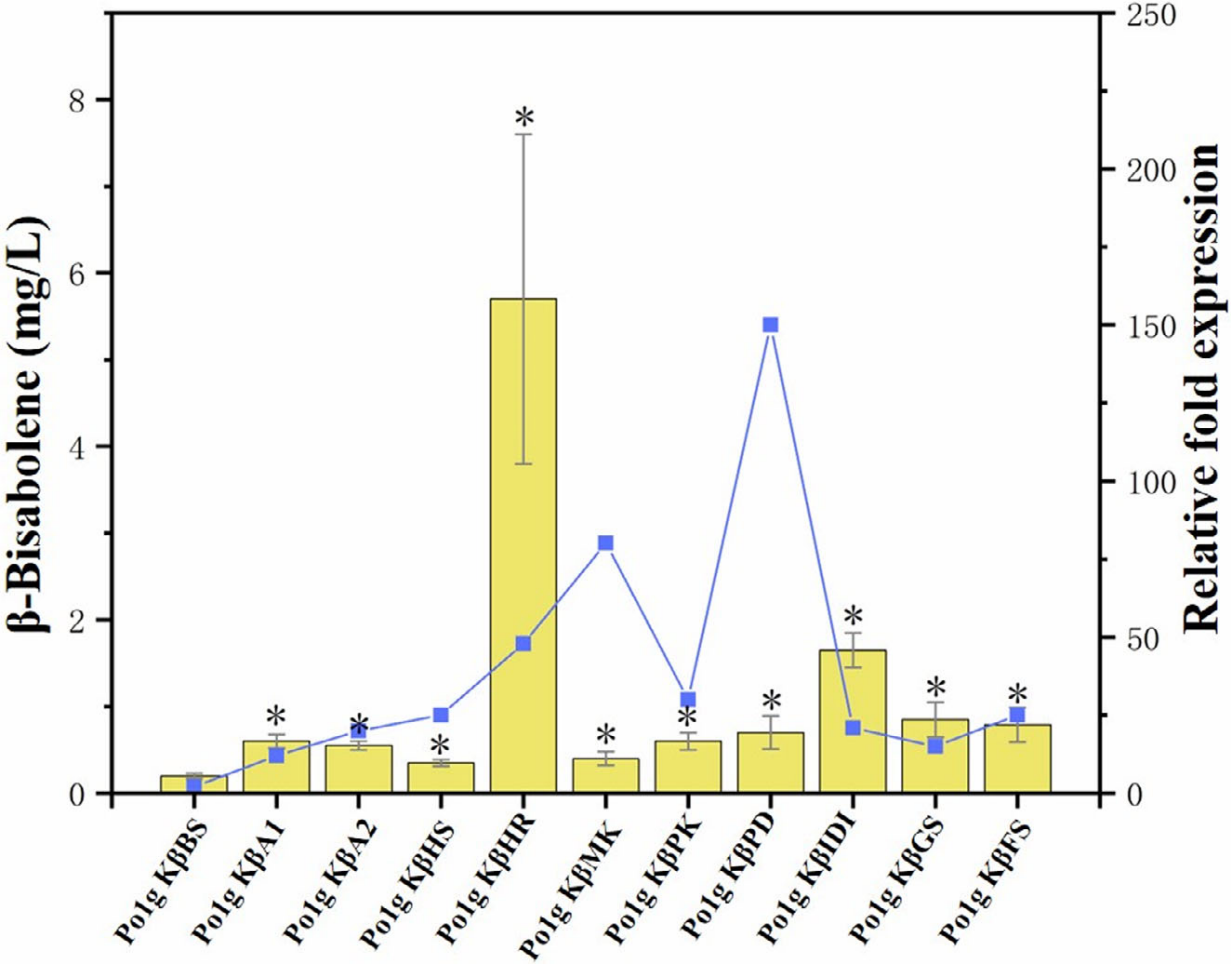
Effects of single‐gene overexpression of genes involved in the MVA pathway on β‐bisabolene production. Ten genes including *ACOAAT1*, *ACOAAT2*, *HMGS*, *HMGR*, *MK*, *PMK*, *PMVADO*, *IPPDI*, *GGPPS* and *FPPS* involved in MVA pathway were overexpressed individually with β‐bisabolene synthase. Bars represent β‐bisabolene titers, and lines represent gene expression increase over controls. Titers of β‐bisabolene were quantified after 5 days of cultivation in shake flasks with 50 ml of liquid YPD medium. The mRNA levels of the ten genes were measured by the SYBR Green I fluorescence method after 3 days of cultivation in shake flasks with 50 ml of liquid YPD medium. Glucose was used as the carbon source. The Po1g KβBS strain cultivated in parallel was used as control, and the gene encoding the *β*‐actin protein was used as the internal standard. Relative gene expression was calculated relative to *β*‐actin by 2^‐▵▵^ *^C^* ^T^ and normalized to controls. All values presented are the mean of three biological replicates ± standard deviation. *P < 0.05, significantly different from control by ANOVA.

**Fig. 4:**
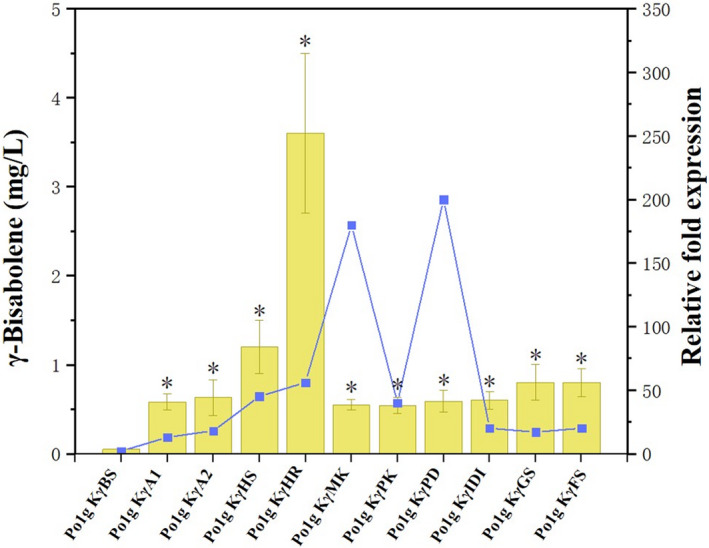
Effects of single‐gene overexpression of genes involved in the MVA pathway on γ‐bisabolene production. Ten genes including *ACOAAT1*, *ACOAAT2*, *HMGS*, *HMGR*, *MK*, *PMK*, *PMVADO*, *IPPDI*, *GGPPS* and *FPPS* involved in MVA pathway were overexpressed individually with γ‐bisabolene synthase. Bars represent γ‐bisabolene titers, and lines represent gene expression increase over controls. Titers of γ‐bisabolene were quantified after 5 days of cultivation in shake flasks with 50 ml of liquid YPD medium. The mRNA levels of the ten genes were measured by the SYBR Green I fluorescence method after 3 days of cultivation in shake flasks with 50 ml of liquid YPD medium. Glucose was used as the carbon source. The Po1g KγBS strain cultivated in parallel was used as control, and the gene encoding the *β*‐actin protein was used as the internal standard. Relative gene expression was calculated relative to *β*‐actin by 2^‐▵▵^ *^C^* ^T^ and normalized to controls. All values presented are the mean of three biological replicates ± standard deviation. *P < 0.05, significantly different from control by ANOVA.

In nature, bisabolene is biosynthesized in plants by bisabolene synthases. Specifically, FPP is produced by the methylerythritol 4‐phosphate (MEP) pathway and converted in the final step of the bisabolene biosynthesis pathway by three different bisabolene synthases into α‐bisabolene, β‐bisabolene and γ‐bisabolene (Fig. 1A). The MEP pathway is absent from *Y. lipolytica*, but this yeast has a native MVA pathway that could supply FPP as substrate (Fig. 1B). Therefore, to construct a complete bisabolene biosynthetic pathway in *Y. lipolytica*, the codon‐optimized genes of α‐bisabolene synthase (α‐BS) from *Abies grandis*, the β‐bisabolene synthase (β‐BS) from *Zingiber officinale* and the γ‐bisabolene synthase (γ‐BS) from *Helianthus annuus* were synthesized and subsequently introduced into the *Y. lipolytica* Po1g KU70Δ strain (Fig. 1B). We selected these three enzyme candidates because they have been successfully applied to produce α‐bisabolene, β‐bisabolene and γ‐bisabolene. The selected α‐BS and β‐BS have already been functionally expressed in *Escherichia coli* (Fujisawa *et al*., 2010; Mcandrew *et al*., 2011), and the γ‐BS chosen has already been functionally expressed in *Saccharomyces cerevisiae* (Aschenbrenner *et al*., 2016). Furthermore, because the rate of precise homologous recombination in the *Y. lipolytica* Po1g KU70Δ strain is much higher than that of the parent strain Po1g, we chose to use Po1g KU70Δ as the starting host strain to facilitate genomic manipulation (Yu *et al*., 2016). Upon integration of the genes of the bisabolene synthases individually into the genome of *Y. lipolytica* for overexpression, the desired compounds were successfully biosynthesized in the recombinant strains (Fig. S1). The resulting engineered strains Po1g KαBS, Po1g KβBS and Po1g KγBS were cultured in the YPD medium. Overlaying with n‐dodecane appears to be the most efficient method to recover the bisabolene production in *Y. lipolytica*, because this method has been proven to be very efficient for trapping large quantities of volatile substances accumulated in different microbes (Willrodt *et al*., 2014; Jongedijk *et al*., 2015; Vickers *et al*., 2015), which may be exported by passive diffusion or active transporters (Chen *et al*., 2013; Liu *et al*., 2015). Addition of dodecane did not show any significant effect on the cell growth of *Y. lipolytica* (Additional file 1: Fig. S2). Thus, a 10% n‐dodecane overlay was added to the culture for capturing the volatile bisabolene. Consequently, the resulting engineered strains Po1g KαBS produced 0.4 mg l^‐1^ of α‐bisabolene (Fig. 2), Po1g KβBS produced β‐bisabolene at 0.2 mg l^‐1^ (Fig. 3) and Po1g KγBS produced 0.05 mg l^‐1^ of γ‐bisabolene (Fig. 4).

### Metabolic engineering of the MVA pathway to improve bisabolene production in Y. lipolytica

With the successful synthesis of bisabolene, we set out to improve the *Y. lipolytica* production of bisabolene via metabolic engineering of the MVA pathway. In the optimization attempt, the genes involved in the MVA pathway were overexpressed to increase the flux towards bisabolene. Ten genes consisting of *ACOAAT1, ACOAAT2*, *HMGS*, *HMGR*, *MK*, *PMK*, *PMVADO*, *IPPDI*, *GGPPS* and *FPPS* were overexpressed, respectively (Fig. 1B), and their effects on the overproduction of bisabolene were examined to determine the rate‐limiting enzyme for bisabolene synthesis in the MVA pathway of *Y. lipolytica*. Thus, a further 30 strains that individually overexpress the ten endogenous genes in the MVA pathway of *Y. lipolytica* were generated based on Po1g KαBS, Po1g KβBS and Po1g KγBS. All genes were integrated into the chromosome and under the control of the strong constitutive promoter hp4d.

Individual overexpression of the selected genes did not have any adverse effect on cell growth (Additional file 1: Fig. S3). Among them, the highest titers were achieved in the *HMGR‐*overexpressing strains Po1g KαHR (Fig. 2), Po1g KβHR (Fig. 3) and Po1g KγHR (Fig. 4), reaching α‐bisabolene, β‐bisabolene and γ‐bisabolene titers of 100.2, 5.7 and 3.6 mg l^‐1^, respectively, after 5 days of culture. These titers correspond to 251‐fold, 29‐fold and 72‐fold enhancement in α‐bisabolene, β‐bisabolene and γ‐bisabolene production, respectively, over the control strains expressing only the respective bisabolene synthases. These results indicate that overexpression of the endogenous 3‐hydroxy‐3‐methylglutaryl coenzyme A (HMG‐CoA) reductase of *Y. lipolytica*, encoded by *HMGR*, is a very efficient way to improve bisabolene biosynthesis in *Y. lipolytica*. This observation is consistent with several other studies where the HMG‐CoA reductase has already been demonstrated to be the key rate‐limiting enzyme in the production of various molecules derived from mevalonate via the MVA pathway (Chappell and Nable, 1987; Stermer and Bostock, 1987; Chappell et al., 1991; Chappell *et al*., 1995; Hampton *et al*., 1996). Therefore, the engineered strains Po1g KαHR, Po1g KβHR and Po1g KγHR were used for subsequent engineering efforts to boost bisabolene production.

### Heterologous expression of two efflux pumps for further enhancement of bisabolene production in Y. lipolytica

Product toxicity is a common problem in strain engineering for biotechnology applications. Small lipophilic products diffuse easily into and through eukaryotic cell membranes, interact with membranes and membrane‐bound enzymes and can also change membrane fluidity and ultrastructure (Stermer and Bostock, 1987; Hampton *et al*., 1996; Parveen *et al*., 2004; Bakkali *et al*., 2008; Witzke *et al*., 2010). Otherwise, they can also cause fungal cells to swell, shrink and vacuolize (Soylu *et al*., 2006). In metabolic engineering, many exogenous compounds, including lipophilic molecules such as bisabolene, are toxic to microorganisms. When designing and engineering metabolic pathways for compound production, undesirable trade‐offs could be introduced because the engineered microorganism must balance production against survival. Cellular export systems, such as efflux pumps, provide a direct mechanism for reducing product toxicity. Studies on ethanol production have shown that alleviating toxicity is necessary to maintain and maximize its production (Alper *et al*., 2006; Jarboe *et al*., 2007).

Effluxing of the compounds produced through metabolic engineering to the extracellular environment is beneficial to the cell factory. Accelerated efflux can alleviate the toxicity associated with the compounds produced and simplify the recovery of target compounds. It has been reported that the use of transport proteins in engineering microorganisms can improve the efficiency of efflux, and the addition of efflux pumps have proved to successfully increase the production of target compounds (Foo and Leong, 2013; Foo et al., 2014; Yoo *et al*., 2019). Furthermore, regardless of toxicity, effective efflux pumps can alleviate the inhibition of metabolic‐pathway enzymes by the products, thereby enhancing the bioproduction of target compounds (Poole, 2001; Wang et al., 2013a,2013b) . With improving production levels, efflux pumps may play an increasingly essential role in enhancing tolerance and production (Dunlop *et al*., 2011). While microorganisms have several strategies for addressing toxicity (Isken and Bont, 1998; Ramos *et al*., 2002), we herein focus on the utilization of efflux pumps, a class of membrane transporters that uses proton motive force or ATP hydrolysis to export toxins from *Y. lipolytica* cells, and thus enhance tolerance to and production of target compounds (Putman *et al*., 2000; Nikaido and Takatsuka, 2009).

For bisabolene extrusion, we selected two transporter candidates. The first is a resistance‐nodulation‐cell division (RND) family efflux pump from *E. coli*, namely, AcrB, because it has been reported that the overexpression of this efflux pump resulted in a 1.5‐fold increase in limonene production in the engineered *E. coli* strain (Dunlop *et al*., 2011). Furthermore, overexpression of this efflux pump also showed an increased tolerance to α‐bisabolene in the engineered *E. coli* strain (Dunlop *et al*., 2011), indicating substrate recognition by AcrB. As the most important part of the AcrAB‐TolC system, to our knowledge, this is the first report of AcrB expression in fungi. The second efflux pump we chose is ABC‐G1 from *Grosmania clavigera*, which is a member of the ATP‐binding cassette (ABC) transporter superfamily. This superfamily is widely present in all five kingdoms of life (Higgins, 1992), and they share a conserved structural architecture and specifically import or export a wide variety of molecules and ions across cellular membranes (Rees *et al*., 2009). The broad polyspecificity of ABC transporters, in general, led us to hypothesize that they could be used to recognize certain terpene molecules and achieve their secretion out of the cell. There is considerable evidence that a wide range of extremely lipophilic molecules can be transported by ABC transporters (Pohl *et al*., 2005), and heterologous expression of ABC‐G1 from *G. clavigera* in *S. cerevisiae* has been shown to increase tolerance to monoterpenes (Wang et al., 2013a,2013b).

**Fig. 5:**
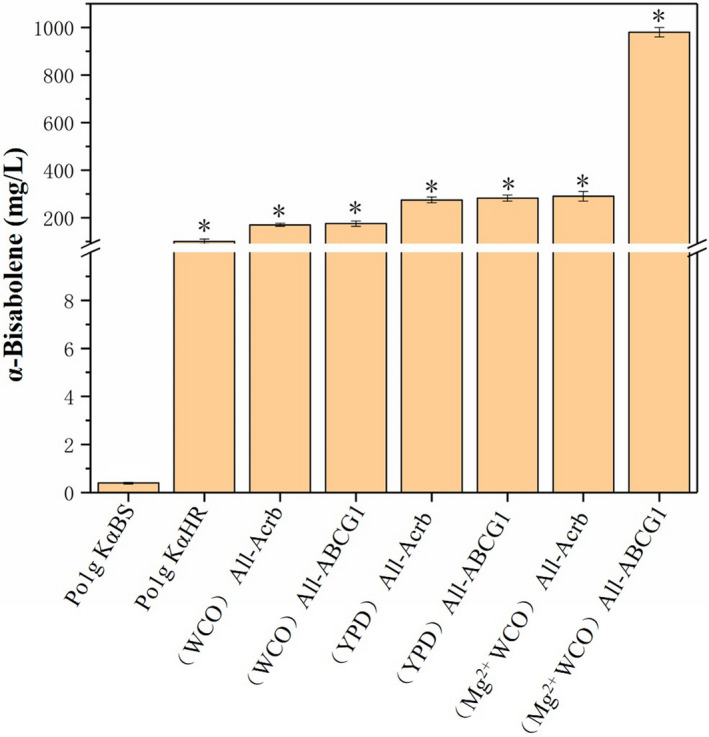
Effect of efflux pump and magnesium ion addition on α‐bisabolene, accumulation during cultures on WCO. The cultivation was performed at 200 rpm with an initial OD_600_ of 0.1 and 10% of n‐dodecane in 50 ml of liquid WCO medium in a 250‐ml shake flask for 5 days. Bars represent bisabolene yields, and lines represent titer fold improvements over controls. Plasmid maps of constructs containing gene integration cassettes were used in this study. The strain all‐Acrb carries the codon‐optimized genes of *HMGR*, *Acrb* and either α‐BS, β‐BS or γ‐BS. The strain all‐ABCG1 carries the codon‐optimized genes of *HMGR*, *ABCG1* and either α‐BS, β‐BS or γ‐BS. All values presented are the mean of three biological replicates ± standard deviation. *P < 0.05, significantly different from control by ANOVA.

**Fig. 6:**
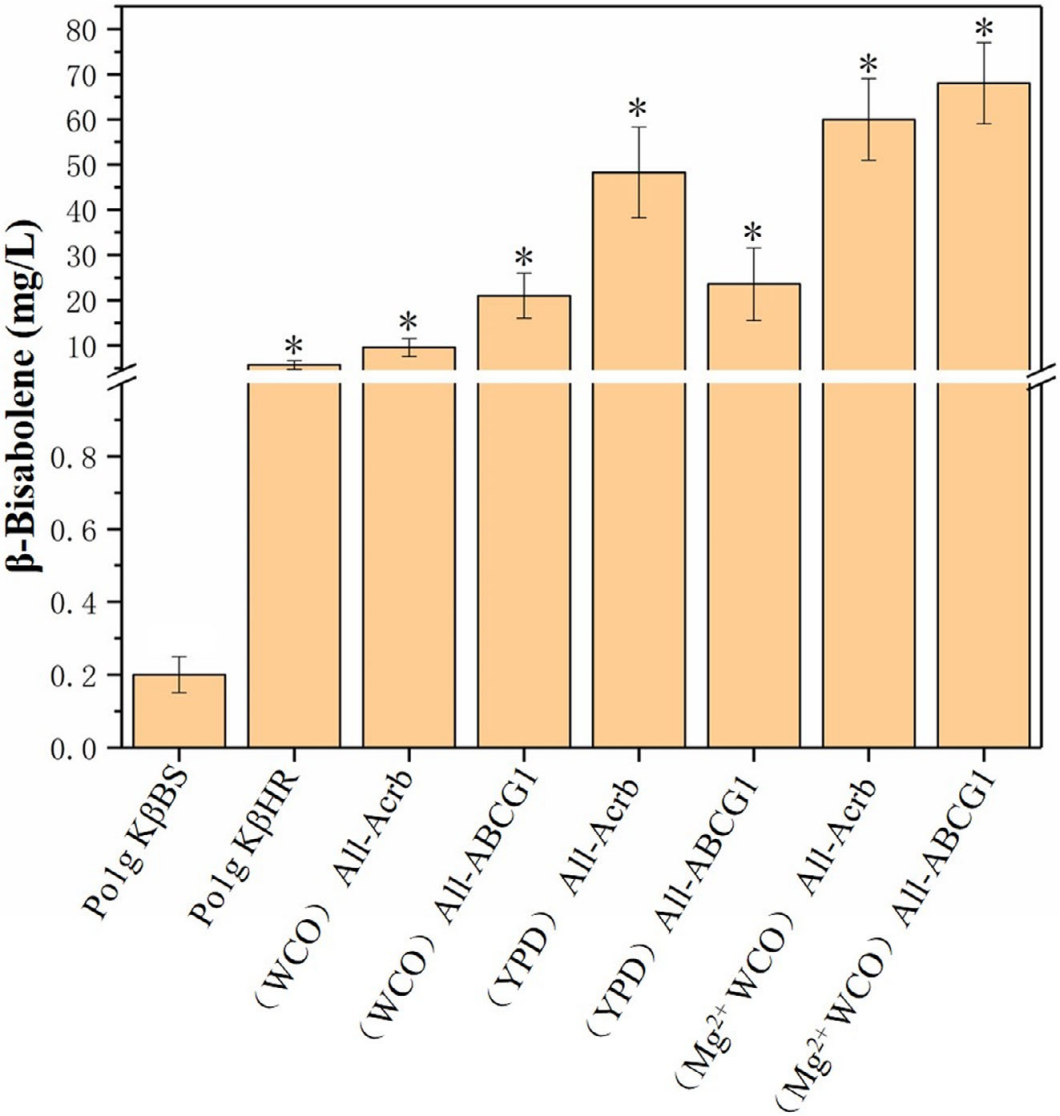
Effect of efflux pump and magnesium ion addition on β‐bisabolene accumulation during cultures on WCO. The cultivation was performed at 200 rpm with an initial OD_600_ of 0.1 and 10% of n‐dodecane in 50 ml of liquid WCO medium in a 250‐ml shake flask for 5 days. Bars represent bisabolene titers, and lines represent titer fold improvements over controls. All values presented are the mean of three biological replicates ± standard deviation. *P < 0.05, significantly different from control by ANOVA.

**Fig. 7:**
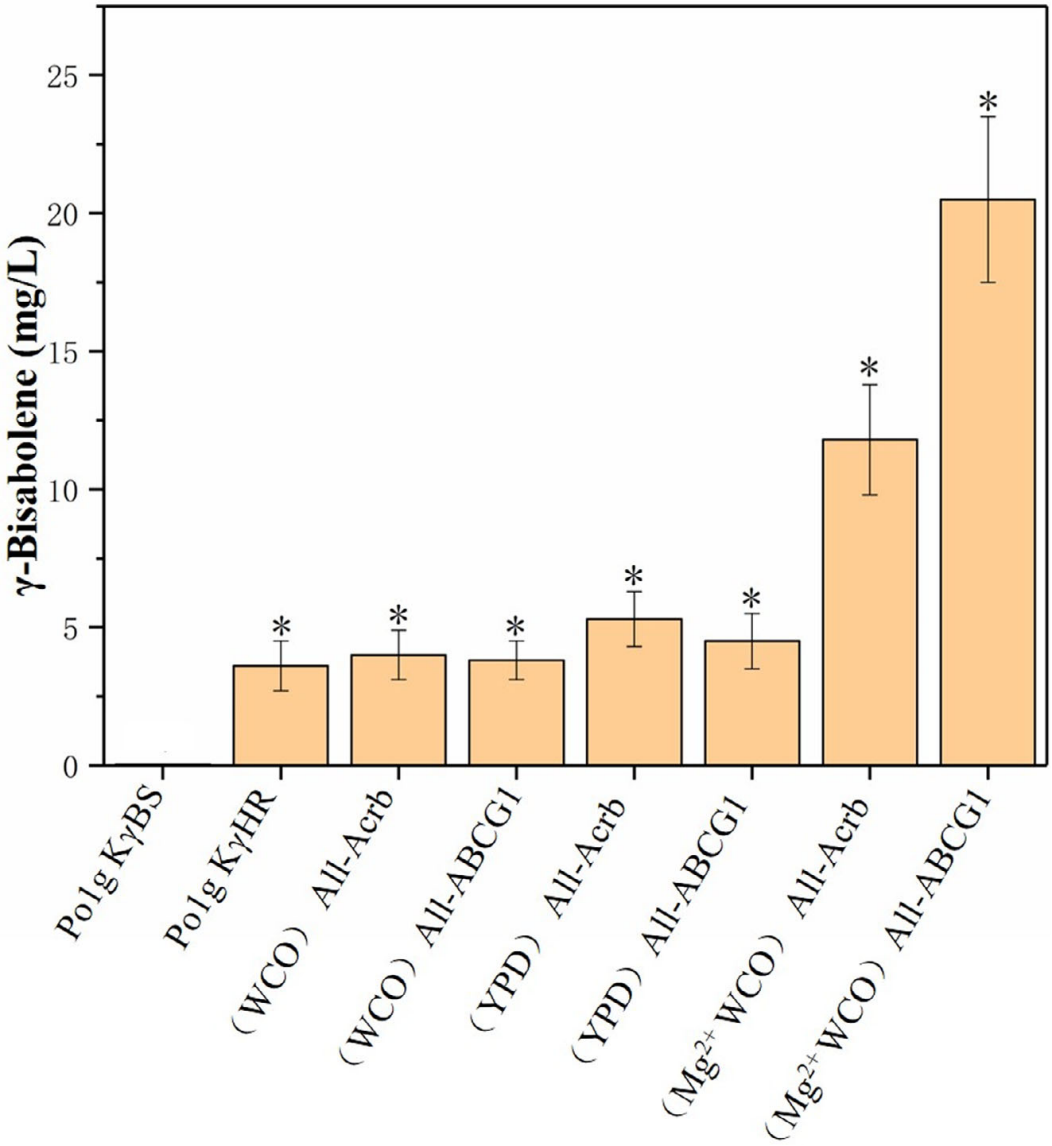
Effect of efflux pump and magnesium ion addition on γ‐bisabolene accumulation during cultures on WCO. The cultivation was performed at 200 rpm with an initial OD_600_ of 0.1 and 10% of n‐dodecane in 50 ml of liquid WCO medium in a 250‐ml shake flask for 5 days. Bars represent bisabolene titers, and lines represent titer fold improvements over controls. All values presented are the mean of three biological replicates ± standard deviation. *P < 0.05, significantly different from control by ANOVA.

To evaluate the effects of the selected transporters on bisabolene production, we heterologously expressed AcrB and ABC‐G1 using the constitutive promoter hp4d in *Y. lipolytica* Po1g KαHR, Po1g KβHR and Po1g KγHR strains respectively. The final engineered strains Po1g KαBS‐AcrB, Po1g KβBS‐AcrB and Po1g KγBS‐AcrB produced 274.4 mg l^‐1^ of α‐bisabolene (Fig. 5), 48.3 mg l^‐1^ of β‐bisabolene (Fig. 6) and 5.3 mg l^‐1^ of γ‐bisabolene (Fig. 7) respectively. These results correspond to 2.7‐fold, 8.5‐fold and 1.2‐fold improvement in α‐bisabolene, β‐bisabolene and γ‐bisabolene titers, respectively, over the respective engineered strains (Po1g KαHR, Po1g KβHR and Po1g KγHR). The engineered strains Po1g KαBS‐ABCG1, Po1g KβBS‐ABCG1 and KγBS‐ABCG1, which overexpressed ABC‐G1, yielded titers of 282.6, 23.6 and 4.3 mg l^‐1^ for α‐bisabolene (Fig. 5), β‐bisabolene (Fig. 6) and γ‐bisabolene (Fig. 7), representing a 2.8‐fold, 4.1‐fold and 1.5‐fold increase in titers as compared with the control strains (Po1g KαHR, Po1g KβHR and Po1g KγHR) respectively. These results validate our hypothesis that the expression of heterologous efflux pumps can boost the production of the different bisabolenes. However, this study only demonstrates the efficacies of the two efflux pumps in improving bisabolene production. Future efforts could be invested in understanding the exact substrate binding and transport mechanisms of the two efflux pumps through structural studies, and subsequent engineering for more efficient and specific efflux pumps for applications in bisabolene production.

### Using waste cooking oils (WCO) as the carbon source for bisabolene production in the engineered Y. lipolytica

Current industrial and domestic practices lead to an excessive production of various low‐value or negative‐cost byproducts and/or crude oil wastes, which may have adverse effects on the environment and human health due to the presence of undesired substances. For example, WCO mainly refers to vegetable oils used at high temperatures in food frying, mixed in kitchen waste and oily wastewater directly discharged into the sewer. Based on the data that 4.1 kg WCO are generated per person per year (Maddikeri *et al*., 2012), it is estimated that the current global annual output of WCO is about 29 million tons (Lisboa *et al*., 2014). Many developed countries have formulated a set of rules that aim to achieve WCO recycling through proper permit transportation, handling and processing. The collected WCO is mainly used as an ingredient in animal feed or as a raw material for biodiesel production. However, WCO still contains undesired substances that could be transferred to humans through the food chain, and for that reason, the European Union has strictly prohibited the recycling of WCO for animal feedstock (Lam *et al*., 2016). Additionally, the presence of polar compounds, impurities, high free fatty acids and water content in WCO might interfere with the biodiesel production and decrease the final quality.

In view of the limitations in using WCO in animal feed and biodiesel production, an alternative approach to recycle WCO is to utilize it as a fermentation media component for the production of value‐added compounds by microorganisms. *Y. lipolytica* is well‐known for its capacity to produce biotechnologically valuable compounds from fatty substrates and agro‐industrial wastes such as olive mill wastewater and crude glycerol (Moftah *et al*., 2013; Ferreira *et al*., 2016). The availability of considerable amounts of WCO at low cost is attractive for ensuring the economic viability of the bioprocesses, while concurrently reducing major environmental issues.

Our group has previously investigated the potential use of WCO as the carbon source for limonene production using engineered *Y. lipolytica* strains. Therefore, we postulate that WCO can also be used as the sole carbon source for bisabolene production in the engineered *Y. lipolytica* constructed in this study. To verify this hypothesis, the strains Po1g KαBS‐AcrB, Po1g KβBS‐AcrB, Po1g KγBS‐AcrB, Po1g KαBS‐ABCG1, Po1g KβBS‐ABCG1 and KγBS‐ABCG1 were cultured by feeding 1.18% (w/v) WCO as the carbon source instead of 2% glucose with the same number of carbon units (information on fatty acid composition of the WCO can be found in Additional file 1: Table S1, Fig. S4). The results show that α‐bisabolene, β‐bisabolene and γ‐bisabolene were successfully produced utilizing WCO as the sole carbon source. The strain Po1g KαBS‐AcrB achieved a titer of 149.9 mg l^‐1^ α‐bisabolene (Fig. 5), and titers of 11.7 and 3.7 mg l^‐1^ for β‐bisabolene and γ‐bisabolene were attained by Po1g KβBS‐AcrB (Fig. 6) and Po1g KγBS‐AcrB (Fig. 7) respectively. The strains Po1g KαBS‐ABCG1, Po1g KβBS‐ABCG1 and KγBS‐ABCG1 produced 157.8 mg l^‐1^ of α‐bisabolene (Fig. 5), 20.9 mg l^‐1^ of β‐bisabolene (Fig. 6) and 3.6 mg l^‐1^ of γ‐bisabolene (Fig. 7) respectively. The OD_600_ achieved by feeding 1.18% WCO was significantly higher than that with 2% glucose (Additional file 1: Fig. S2), demonstrating that our engineered bisabolene‐producing *Y. lipolytica* strains can grow very efficiently and robustly on WCO as the sole carbon source. Although n‐dodecane is a known carbon source for *Y. lipolytica*, it did not contribute significantly to the growth of our engineered strains in the rich medium that we used for cultivation (Additional file 1: Fig. S2). However, the titers of α‐bisabolene, β‐bisabolene and γ‐bisabolene achieved in WCO medium declined compared with YPD medium, which contains glucose as the carbon source. This result can be attributed to the suboptimal cultivation parameters (e.g. concentration of ions, pH and dissolved oxygen level) for bisabolene accumulation of the engineered *Y. lipolytica* strains in this original culture. Therefore, further optimization is required to overcome these hurdles. The outcome of this study suggests that our engineered *Y. lipolytica* strains can serve as a platform strain for future metabolic engineering efforts to biosynthesize bisabolene and valuable bisabolene‐derived chemicals from WCO.

### Adding magnesium ion further enhances bisabolene production

Metal ions are important factors affecting the growth of and metabolite biosynthesis in microorganisms. They participate in many biological processes, including regulating enzyme activity, maintaining the stability of biological macromolecules and cell structures, regulating the balance of cell osmotic pressure and controlling the redox potential of cells (Xiang *et al*., 2010). The level of metal ions can also affect the functions of transcription factors and have certain impact on the growth microenvironment of cells (Zhao *et al*., 2013).

Among the metal ions, magnesium ion is a cofactor of several important enzymes, including pyruvate decarboxylase, pyruvate kinase, hexokinase, phosphofructokinase, glucose‐6‐phosphate dehydrogenase, citrate lyase and isocitrate dehydrogenase. These enzymes play essential roles in regulating glycolysis, respiration, oxidative phosphorylation and other processes. For oleaginous microorganisms such as *Y. lipolytica*, magnesium ions can bind to the key enzymes in lipid synthesis by affecting the structural integrity of the enzymes. Jernejc and Legisa (2002) added magnesium ions to *Aspergillus niger* and found that the activity of malic enzyme was increased, and thereby, the formation of reducing power (NADPH) was enhanced. Previously, it was also proven that the addition of Mg^2+^ has great influence on fermentation performance of oleaginous microorganisms.

Previously, our group has determined that the addition of Mg^2+^ could effectively improve the production of d‐limonene and l‐limonene in the engineered *Y. lipolytica*. First, the effect of Mg^2+^ on growth of *Y. lipolytica* was investigated, and the results suggested that the effect of exogenous magnesium on the growth of the engineered *Y. lipolytica* strains was not significant under our experimental conditions (Fig. S5).

In addition, the optimal fermentation parameters for limonene production by the *Y. lipolytica* strains were already established. They are as follows: a temperature of 20℃, a rotation speed of 250 rpm, pH 5.74 and 0.2% Mg^2+^ (Pang *et al*., 2019). These conditions were applied in a typical batch fermentation of the strains overexpressing the ABC‐G1 pump, which resulted in the highest production of 973.1 mg l^‐1^ of α‐bisabolene (Fig. 5), 68.2 mg l^‐1^ of β‐bisabolene (Fig. 6) and 20.2 mg l^‐1^ of γ‐bisabolene (Fig. 7), representing 2433‐, 341‐ and 404‐fold increase over that of the starting strains respectively. Similarly, the strains overexpressing the Acrb pump were fermented, and the titers improved to varying degrees. α‐Bisabolene production increased 713‐fold to achieve a titer of 285.2 mg l^‐1^, β‐bisabolene production increased 297‐fold to achieve a titer of 59.3 mg l^‐1^ and γ‐bisabolene production increased 222‐fold to achieve a titer of 11.1 mg l^‐1^ as shown in Figs 5, 6, 7. Thus, here we demonstrated that addition of Mg^2+^ could also lead to increased titers of bisabolene in the engineered *Y. lipolytica*, which is similar to the case of limonene (Pang *et al*., 2019). The improved production could possibly be due to Mg^2+^ enhancing the activities of enzymes in the MVA pathway or bisabolene synthases in the engineered *Y. lipolytica* strains, which has been reported in the literatures (Colby *et al*., 1993; Maruyama *et al*., 2001). In future work, more studies will be needed for better understanding of the mechanisms behind the beneficial effect of Mg^2+^ on the production of both bisabolene and limonene in *Y. lipolytica*.

## Discussion

Bisabolene is in great demand due to its wide range of industrial applications. The metabolic engineering of microorganisms provides a platform for the effective production of these valuable compounds. Here, we used metabolic engineering tools to construct a new pathway in *Y. lipolytica* to enzymatically convert the abundant acetyl‐CoA pool in the oleaginous yeast host into α‐bisabolene, β‐bisabolene and γ‐bisabolene. To our knowledge, we have unprecedentedly verified the function of α‐bisabolene synthase, β‐bisabolene synthase and γ‐bisabolene synthase in *Y. lipolytica* and successfully employed the enzymes to synthesize bisabolene in *Y. lipolytica* for the first time. It is worth noting that the differences in gene expression levels of the heterologous bisabolene synthases from three different sources were slight (Figs 2, 3, 4), but the production levels of bisabolene were significantly different. The different yields of bisabolene isomers may be affected by the translational efficiency of the genes and inherent enzyme activity; more bisabolene synthase genes from different sources can be screened in future to construct high‐yielding systems.

Next, we overexpressed efflux pumps and proved that it correlated with a moderate increase in the production of bisabolene. Our study reports, for the first time, the utilization of terpenoid efflux pumps to improve the secretion and thus the production of bisabolene in microbial cell factories. Subsequently, we investigated the potential of our engineered bisabolene‐producing *Y. lipolytica* strains in using WCO to produce bisabolene. We demonstrated for the first time the conversion of waste cooking oil to bisabolene, which has important potential applications in waste management as well as economical and sustainable production of valuable bisabolene from waste feedstocks. In addition, we also found that the supplementation of Mg^2+^ can greatly increase the titer of bisabolene in the engineered *Y. lipolytica* strains. Finally, the highest titers of α‐bisabolene, β‐bisabolene and γ‐bisabolene achieved from WCO in the engineered *Y. lipolytica* strains were 973.1 mg l^‐1^ for α‐bisabolene, 68.2 mg l^‐1^ for β‐bisabolene and 20.2 mg l^‐1^ for γ‐bisabolene. These titers correspond to 2433‐fold, 340‐fold and 404‐fold enhancement in bisabolene production, respectively, over the starting strains. These results demonstrate the efficacy of the combinatorial engineering strategies applied for the production of bisabolene in *Y. lipolytica* in this study.

To date, there are several studies on successful production of bisabolene by metabolic engineering of microbes (Table 1). Conventional microbial hosts (i.e. *E. coli* and *S. cerevisiae*) so far have been the most widely used workhorses for metabolic engineering towards bisabolene production owing to their ease of genetic manipulation. Compared with the available data from previous studies, the final titer of α‐bisabolene that we achieved in *Y. lipolytica* is comparable with the highest reported titer in *E. coli*. Although the reported titer of α‐bisabolene in *S. cerevisiae* is higher than that achieved in this work, many more genetic modifications were performed in the engineered *S. cerevisiae* (Peralta‐Yahya *et al*., 2011; Ӧzaydın *et al*., 2013). We envision that in the future higher α‐bisabolene titer could be achieved in *Y. lipolytica* through more extensive metabolic engineering.

**Table 1:** Bisabolene production in the metabolically engineered microbial hosts.

Host	Product	Titer	Yield	Productivity	Strategy	Reference
*Y. lipolytica*	α‐bisabolene	973.1 mg l^‐1^	–	8.11 mg l^‐1^ h^‐1^	Producing α‐bisabolene, β‐bisabolene and γ‐bisabolene through heterologous expression of the α‐bisabolene synthase from *A. grandis*, the β‐bisabolene synthase gene from *Z. officinale* and the γ‐bisabolene synthase gene from *H. annuus* respectively. Subsequently, overexpression of the endogenous mevalonate pathway genes and introduction of heterologous multidrug efflux transporters. Furthermore, the fermentation conditions were optimized to maximize bisabolene production by the engineered *Y. lipolytica* strains from glucose	In this study
	β‐bisabolene	68.2 mg l^‐1^	–	0.56 mg l^‐1^ h^‐1^		
	γ‐bisabolene	20.2 mg l^‐1^	–	0.17 mg l^‐1^ h^‐1^		
*E. coli*		α‐bisabolene	912 mg l^‐1^	182.4 mg g glucose^‐1^	12.49 mg l^‐1^ h^‐1^	The heterologous codon‐optimized version of the highest α‐bisabolene synthase gene *Ag1* from *A. grandis* was coexpressed with four homologous codon‐optimized genes *tHMGR*, *HMGS*, *MK* and *PMK* involved in the MVA pathway from *S. cerevisiae* under control of a strong promoter Ptrc	(Peralta‐Yahya *et al*., 2011)
		bisabolene	1.1 g l^‐1^	–	15.28 mg l^‐1^ h^‐1^	Inducer‐free bisabolene production was achieved by expressing LuxR/LuxI effector‐regulator proteins and using PluxI responsive promoter to drive target biosynthesis pathway with a QS system	(Kim *et al*., 2017)
*S. cerevisiae*		α‐bisabolene	994 mg l^‐1^	–	10.35 mg l^‐1^ h^‐1^	The heterologous codon‐optimized version of the highest α‐bisabolene synthase gene *Ag1* from *A. grandis* was coexpressed with the truncated HMG‐CoA reductase (tHMGR), the FPP synthase (Erg20), and the global transcription regulator of the sterol pathway upc2‐1 and the squalene synthase (Erg9) was downregulated	(Peralta‐Yahya *et al*., 2011)
		bisabolene	5.2 g l^‐1^	250 mg g glucose^‐1^	217 mg l^‐1^ h^‐1^	Introducing gene deletions (*YJL064W*, *YPL062W* and *ROX1*) into strains. Overexpressed *tHMG1* and *ERG20* along with the *AgBIS* and downregulated *ERG9*	(Ӧzaydın *etal*., 2013)
*Synechococcus* sp. PCC 7002		α‐bisabolene	0.6 mg l^‐1^	–	6.25 µg l^‐1^ h^‐1^	The heterologous *A. grandis* α‐bisabolene synthase gene *Ag1* was expressed.	(Davies *et al*., 2014)
*Synechocystis* sp. PCC 6803		α‐bisabolene	22.2 mg l^‐1^	–	0.03 mg l^‐1^ h^‐1^	Improving heterologous protein expression in *Synechocystis* sp. PCC 6803 by combining RBS calculator and codon optimizations under light condition	(Sebesta and Peebles, 2019)
*Chlamydomonas reinhardtii*		α‐bisabolene	11 mg l^‐1^	–	0.07 mg l^‐1^ h^‐1^	Combining sequential enzyme loading and amiRNA knockdown from four separate genetic constructs and using different carbon and light regimes	(Wichmann *et al*., 2017)
*Rhodosporidium toruloides*		bisabolene	680 mg l^‐1^	–	5.04 mg l^‐1^ h^‐1^	Growing in corn stover hydrolysates prepared by two different pretreatment methods, one using a novel biocompatible ionic liquid (IL) choline α‐ketoglutarate at bench scale, and the other using an alkaline pretreatment in a high‐gravity fed‐batch bioreactor	(Yaegashi *et al*., 2017)

As described above, this study shows that bisabolene can be efficiently produced by employing metabolically engineered *Y. lipolytica*. Notably, we demonstrated that the engineered *Y. lipolytica* strains are highly promising microbial platforms for converting WCO into the valuable sesquiterpene and its derivatives, which will bring major breakthroughs to waste conversion and the biochemical industry. We conclude that metabolic engineering will continue to play key roles in developing such economically competitive bioprocesses. However, *Y. lipolytica* as a platform with great potential in converting WCO into bisabolene needs to be further engineered to obtain higher production of bisabolene to realize full‐scale commercialization and industrialization. In our future research, metabolic engineering strategies for further reinforcing the metabolic flux of MVA pathway towards FPP, reducing the flux of competing pathways and discovery or engineering of bisabolene synthases with improved catalytic activity could be performed to enhance bisabolene production in engineered *Y. lipolytica*.

## Experimental procedures

All chemicals, solvents and media components, were purchased and used without modification. *Pml* Ⅰ, *Kpn* Ⅰ, *Spe* Ⅰ, *Nru* Ⅰ and *Hpa* Ⅰ were purchased from New England Biolabs (Beverly, MA, USA), ClonExpress ^®^Ⅱ one step cloning kit, 2 × Rapid taq master mix and 2 × Phanta ^®^ max master mix were purchased from Vazyme Biotech Co., Ltd. (Nanjing, China), peptone and yeast extract were purchased from Thermo Scientific Oxoid Microbiology Products (Basingstoke, UK), n‐dodecane was purchased from Aladdin ^®^ (Los Angeles, CA, USA), bisabolenes were purchased from Sigma‐Aldrich (St. Louis, MI, USA), DNA salmon sperm, plasmid eExtraction mini kits and DNA purification kits were purchased from Solarbio life sciences (Beijing, China). *E. coli* DH5α was used for plasmid construction and amplification, and *E. coli* strains were routinely cultured in LB medium supplemented with 100 μg ml^‐1^ of ampicillin at 37°C. *Y. lipolytica* Po1g KU70Δ was used as the base strain in this study. Routine cultivation of *Y. lipolytica* strains was carried out at 30°C in YPD medium. In experiments employing WCO as the carbon source, 2% glucose was removed and replaced with appropriate concentrations of WCO. WCO was collected from a local kitchen. The growth medium was also supplemented with Tween‐80 when WCO was used as the carbon source. All of the recombinant plasmids were constructed using the One Step Cloning Kit from Vazyme Biotech Co., Ltd (Nanjing, China). Plasmids used in this study are listed in Table S2, and strains are listed in Table S3. PCR primers used in this study were synthesized by Genewiz (San Diego, CA, USA) and listed in Table S4.

### Strains, vectors and culture conditions

*Y. lipolytica* Po1g KU70Δ was used as the base strain in this study. Routine cultivation of *Y. lipolytica* strains was carried out at 30°C in YPD medium. *E. coli* DH5α was used for plasmid construction and amplification, and *E. coli* strains were routinely cultured in LB medium supplemented with 100 μg ml^‐1^ of ampicillin at 37°C. The yeast extract peptone dextrose (YPD) medium (20 g l^‐1^ glucose, 20 g l^‐1^ peptone, and 10 g l^‐1^ yeast extract) was used for strain activation; whereas, the yeast synthetic completely (YNB) (6.7 g l^‐1^ yeast nitrogen base without amino acids, 20 g l^‐1^ glucose, 15 g l^‐1^ Bacto agar) lacking the appropriate nutrients was used for the screening of transformants.

### Plasmids construction and yeast transformation

The α‐bisabolene synthase gene (*αBS*, GenBank ID: AF006195.1) from *A. grandis*, β‐bisabolene synthase gene (*βBs*, GenBank ID: AB511914.1) from *Z. officinale* and γ‐bisabolene synthase gene (*γBS*, GenBank ID: KU674381.1) from *H. annuus* were synthesized and codon‐optimized by Genewiz. The genes *αBS, βBs* and *γBS* were cloned into pYLEX1 (Fig. S6) with primers α‐F/α‐R, β‐F/β‐R and γ‐F/γ‐R (Additional file 1: Table S4) to yield plasmids pYLαBS, pYLβBS and pYLγBS respectively. The genes *ACOAAT1*, *ACOAAT2*, *HMGS*, *HMGR*, *MK*, *PMK*, *PMVADO*, *IPPDI*, *GGPPS* and *FPPS* were cloned into pYLEX1 with primers ACOAAT1‐F/ACOAAT1‐R, ACOAAT2‐F/ ACOAAT2‐R, HMGS‐F/HMGS‐ R, HMGR‐F/HMGR‐R, MK‐F/MK‐R, PMK‐F/PMK‐R, PMVADO‐F/PMVADO‐R, IPPDI‐F/IPPDI‐R, GGPPS‐ F/GGPPS‐R and FPPS‐F/FPPS‐R to yield plasmids pYLA1, pYLA2, pYLHS, pYLHR, pYLMK, pYLPK, pYLPD, pYLIDI, pYLGS and pYLFS respectively. The expression cassettes of *ACOAAT1*, *ACOAAT2*, *HMGS*, *HMGR*, *MK*, *PMK*, *PMVADO*, *IPPDI*, *GGPPS* and *FPPS* were cloned into pYLαBS, pYLβBS and pYLγBS with primers BDH‐F/BDH‐R to yield plasmids pYLαA1, pYLαA2, pYLαHS, pYLαHR, pYLαMK, pYLαPK, pYLαPD, pYLαIDI, pYLαGS, pYLαFS, pYLβA1, pYLβA2, pYLβHS, pYLβHR, pYLβMK, pYLβPK, pYLβPD, pYLβIDI, pYLβGS, pYLβFS, pYLγA1, pYLγA2, pYLγHS, pYLγHR, pYLγMK, pYLγPK, pYLγPD, pYLγIDI, pYLγGS and pYLγFS respectively.

The AcrB efflux pump gene from *E. coli* and the ABC‐G1 efflux pump gene from *G. clavigera* were synthesized and codon‐optimized by Genewiz. The expression cassettes of AcrB and ABC‐G1 were cloned into pYLαHR, pYLβHR and pYLγHR with primers Acrb‐BDH‐F/Acrb‐BDH‐R and CMQ‐BDH‐F/CMQ‐BDH‐R to yield plasmids pYLαHR‐Acrb, pYLβHR‐Acrb, pYLγHR‐Acrb, pYLαHR‐CMQ, pYLβHR‐CMQ and pYLγHR‐CMQ respectively. All plasmids were first linearized with *Spe* I and then transformed into competent cells of *Y. lipolytica* strains using the lithium acetate/single‐stranded carrier DNA/polyethylene glycol method (Yu *et al*., 2016).

### Strain construction

Yeast colonies of *Y. lipolytica* Po1g KU70Δ were grown in 50 ml of fresh YPD medium for 24 h. Cells were pelleted and washed twice with 20 ml Tris‐EDTA (TE) buffer (10 mM Tris, 1 mM EDTA, pH 7.5) and once with 0.1 M lithium acetate (pH 6.0). The cells were then re‐suspended with 5 ml of 0.1 M lithium acetate (pH 6.0) and incubated for 10 min at room temperature. An aliquot of 100 μl competent cells was placed into sterile 2 ml tubes. The competent cells were then mixed by vortexing with 0.7 ml of 40% PEG‐4000, 10 μl of denatured salmon sperm DNA and 10 μl of linearized recombination plasmids for 1 h. The transformation mixture was incubated at 39°C for 1 h. Then, add 1 ml YPD medium and recover for 2 h at 30°C and 225 rpm. Following that, the transformation mixture was pelleted, re‐suspended in water and plated directly onto YNB plates. After selection, the following engineered *Y. lipolytica* strains were generated: Po1g KαBS, Po1g KβBS, Po1g KγBS, Po1g KαA1, Po1g KαA2, Po1g KαHS, Po1g KαHR, Po1g KαMK, Po1g KαPK, Po1g KαPD, Po1g KαIDI, Po1g KαGS, Po1g KαFS, Po1g KβA1, Po1g KβA2, Po1g KβHS, Po1g KβHR, Po1g KβMK, Po1g KβPK, Po1g KβPD, Po1g KβIDI, Po1g KβGS, Po1g KβFS, Po1g KγA1, Po1g KγA2, Po1g KγHS, Po1g KγHR, Po1g KγMK, Po1g KγPK, Po1g KγPD, Po1g KγIDI, Po1g KγGS, Po1g KγFS, Po1g KαHR‐Acrb, Po1g KαHR‐CMQ, Po1g KβHR‐Acrb, Po1g KβHR‐CMQ, Po1g KγHR‐Acrb and Po1g KγHR‐CMQ (Table S3).

### Culturing the engineered Y. lipolytica strains for α‑bisabolene, β‑bisabolene and γ‑bisabolene production

To produce α‑bisabolene, β‑bisabolene and γ‑bisabolene, seed cultures were prepared by inoculating 5 ml of YPD medium in the 20‐ml culture tubes with the engineered *Y. lipolytica* strains. The cells were grown at 30°C for 24 h with agitation. Following that, 250‐ml flasks containing 25 ml of YPD medium were inoculated to OD_600_ 0.1 with the seed cultures. All cultures were shaken at 200 rpm and 30°C. To avoid loss of bisabolene during cultivation, 10% of *n*‐dodecane overlay was added into the YPD medium prior to cultivation. Samples were then collected at day 5. In order to further increase the yields of α‑bisabolene, β‑bisabolene and γ‑bisabolene, WCO was added to the YPD medium as a carbon source to replace glucose with the same carbon number. Using the optimum fermentation parameters determined before, 0.2% Mg^2+^ was added to the medium, and the *n*‐dodecane layer was added into WCO medium before fermentation. At the end of fermentation, the mixture of the *n*‐dodecane layer and WCO formed a one‐layer system after centrifuging at 7500 rpm for 10 min. Therefore, the upper one‐layer organic phase was used for analysis of bisabolene production by GC/MS.

### GC–MS analysis of α‑bisabolene, β‑bisabolene and γ‑bisabolene produced in the engineered Y. lipolytica strains

For the determination of α‑bisabolene, β‑bisabolene and γ‑bisabolene, all cultures were centrifuged at 7500 rpm for 10 min at each time of sampling. Specially, in the experiments using WCO as the sole carbon source, this volume of organic phase analysed by GC–MS represents the total volume of WCO and *n*‐dodecane. Then, 1 μl organic phase was analysed by GC–MS using an Agilent 7890A GC with an 5975C MSD equipped with a HP‐5MS column (60 m × 0.25 mm × 0.25 μm; Agilent, Santa Clara, CA, USA). GC oven temperature was initially held at 60°C for 2 min, and then ramped to 140°C at a rate of 5°C min^‐1^. It was then subsequently ramped at 5°C min^‐1^ to 280°C and held for 5 min. The split ratio was 10:1. Helium was used as the carrier gas, with an inlet pressure of 13.8 psi. The injector was maintained at 280°C, and the ion source temperature was set to 230°C. Final data analysis was achieved using Enhanced Data Analysis software (Agilent, Santa Clara, CA, USA).

### Statistical analysis

Differences in titers between the control strain and other strains were evaluated using SPSS 22.0 software for Windows (SPSS, Chicago, IL, USA). One‐way ANOVA analyses were carried out with a confidence interval of 95%, and statistical significance was considered if *P* < 0.05. All assays were performed at least in triplicate and reported as mean ± SEM. * indicates the groups that were significantly different from the relevant control cells.

## Conflict of interests

The authors declare that they have no competing interests.

## Funding Information

The Natural Science Foundation of Tianjin, China (17JCYBJC40800), the Research Foundation of Tianjin Municipal Education Commission, China (2017ZD03), the Innovative Research Team of Tianjin Municipal Education Commission, China (TD13‐5013), Tianjin Municipal Science and Technology Project (18PTSYJC00140, 19PTSYJC00060), the Open Project Programme of State Key Laboratory of Food Nutrition and Safety (SKLFENS‐KF‐201915), Startup Fund for ‘Haihe Young Scholars’ of Tianjin University of Science and Technology, the Thousand Young Talents Programme of Tianjin, China.

## Authors' contributions

AQY, DGX and CYZ conceived and designed the study. YKZ, KZ, JL, YZ and SLL performed plasmid and strain construction and fermentation experiments. YKZ and JL wrote the manuscript. AQY, DGX and CYZ revised the manuscript. All authors read and approved the final manuscript.

## Ethics approval and consent to participate

This manuscript does not contain any studies with human participants or animals performed by any of the authors.

## Consent for publication

All authors give consent to publish the research in Microbial Biotechnology.

## Supporting information


**Fig. S1**. GC–MS profile of solvent overlay‐extracted bisabolenes from cultures of engineered *Y. lipolytica* strains.
**Fig. S2**. Effect of different carbon sources on growth of *Y. lipolytica* Po1g KαBS‐ABCG1.
**Fig. S3**. The OD_600_ values of 30 engineered *Y. lipolytica* strains cultured in YPD medium.
**Fig. S4**. The GC–MS analysis of fatty acid composition in waste cooking oil.
**Fig. S5**. Effect of Mg^2+^ on growth of *Y. lipolytica*.
**Fig. S6**. Map of the plasmid pYLEX1.
**Table S1**. Information on fatty acid composition of the waste cooking oil.
**Table S2**. Plasmids used in this study.
**Table S3**. Strains used in this study.
**Table S4**. Plasmids used in this study. Primers used in PCR.

## Data Availability

All relevant data generated or analysed during this study were included in this published article.
