# Supplementary material for: High‐efficiency production of bisabolene from waste cooking oil by metabolically engineered Yarrowia lipolytica
Source: Microb Biotechnol. 2021 Feb 19;14(6):2497–513. doi: 10.1111/1751-7915.13768 (PMC8601197; doi:10.1111/1751-7915.13768)
Supplement: Supplementary file 1 — Fig. S1. GC–MS profile of solvent overlay‐extracted bisabolenes from cultures of engineered Y. lipolytica strains. Fig. S2. Effect of different carbon sources on growth of Y. lipolytica Po1g KαBS‐ABCG1. Fig. S3. The OD600 values of 30 engineered Y. lipolytica strains cultured in YPD medium. Fig. S4. The GC–MS analysis of fatty acid composition in waste cooking oil. Fig. S5. Effect of Mg2+ on growth of Y. lipolytica. Fig. S6. Map of the plasmid pYLEX1. Table S1. Information on fatty acid composition of the waste cooking oil. Table S2. Plasmids used in this study. Table S3. Strains used in this study. Table S4. Plasmids used in this study. Primers used in PCR. [file MBT2-14-2497-s001.docx]

**SUPPLEMENTARY INFORMATION**

**High-efficiency production of bisabolene from waste cooking oil by metabolically engineered *Yarrowia lipolytica***

Yakun Zhao^a^, Kun Zhu^a^, Jian Li^a^, Yu Zhao^a^, Shenglong Li^a^, Cuiying Zhang^a^, Dongguang Xiao^a^, Aiqun Yu^a*^

**Table S1 Information** **on fatty acid composition of the waste cooking oil used in this study**

| Fatty acid | Waste cooking oil (g/100g) |
| --- | --- |
| C8:0 | 0.0406 |
| C14:0 | 0.0520 |
| C15:0 | 0.0021 |
| C16:0 | 9.1128 |
| C16:1 | 0.0592 |
| C17:0 | 0.0704 |
| C17:1 | 0.0286 |
| C18:0 | 3.5801 |
| C18:1n9t | 0.0953 |
| C18:1n9c | 18.8678 |
| C18:2n6c | 48.3580 |
| C20:0 | 0.3847 |
| C18:3n6 | 0.6781 |
| C20:1 | 0.3990 |
| C18:3n3 | 5.6221 |
| C21:0 | 0.0386 |
| C20:2 | 0.0492 |
| C22:0 | 0.5505 |
| C23:0 | 0.0616 |
| C20:5n3 | 0.5014 |
| C24:1 | 0.0364 |

Waste cooking oil was collected from the canteen of Tianjin University of Science and Technology, and the fatty acids of commercial vegetable oil and waste cooking oil were detected by Qingdao Sci-tech Innovation Quality Testing Co., Ltd.

**Table S2** **Plasmids used in this study**

| Plasmid | Features | Reference |
| --- | --- | --- |
| pYLEX1 | *Y. lipolytica*‑integrative plasmid, P_hp4d_-T_XPR2_, LEU2 | (Madzak *et al.,* 2000) |
| pYLαBS | P_hp4d_-αBS-T_XPR2_, LEU2 | This study |
| pYLβBS | P_hp4d_-βBS-T_XPR2_, LEU2 | This study |
| pYLγBS | P_hp4d_-γBS-T_XPR2_, LEU2 | This study |
| pYLA1 | P_hp4d_-A1-T_XPR2_, LEU2 | This study |
| pYLA2 | P_hp4d_-A2-T_XPR2_, LEU2 | This study |
| pYLHS | P_hp4d_-HS-T_XPR2_, LEU2 | This study |
| pYLHR | P_hp4d_-HR-T_XPR2_, LEU2 | This study |
| pYLMK | P_hp4d_-MK-T_XPR2_, LEU2 | This study |
| pYLPK | P_hp4d_-PK-T_XPR2_, LEU2 | This study |
| pYLPD | P_hp4d_-PD-T_XPR2_, LEU2 | This study |
| pYLIDI | P_hp4d_-IDI-T_XPR2_, LEU2 | This study |
| pYLGS | P_hp4d_-GS-T_XPR2_, LEU2 | This study |
| pYLFS | P_hp4d_-FS-T_XPR2_, LEU2 | This study |
| pYLαA1 | P_hp4d_-αBS-T_XPR2_, P_hp4d_-A1-T_XPR2_, LEU2 | This study |
| PYLαA2 | P_hp4d_-αBS-T_XPR2_, P_hp4d_-A2-T_XPR2_, LEU2 | This study |
| pYLαHS | P_hp4d_-αBS-T_XPR2_, P_hp4d_-HS-T_XPR2_, LEU2 | This study |
| pYLαHR | P_hp4d_-αBS-T_XPR2_, P_hp4d_-HR-T_XPR2_, LEU2 | This study |
| pYLαMK | P_hp4d_-αBS-T_XPR2_, P_hp4d_-MK-T_XPR2_, LEU2 | This study |
| pYLαPK | P_hp4d_-αBS-T_XPR2_, P_hp4d_-PK-T_XPR2_, LEU2 | This study |
| pYLαPD | P_hp4d_-αBS-T_XPR2_, P_hp4d_-PD-T_XPR2_, LEU2 | This study |
| pYLαIDI | P_hp4d_-αBS-T_XPR2_, P_hp4d_-IDI-T_XPR2_, LEU2 | This study |
| pYLαGS | P_hp4d_- dLS -T_XPR2_, P_hp4d_-GS-T_XPR2_, LEU2 | This study |
| pYLαFS | P_hp4d_-αBS-T_XPR2_, P_hp4d_-FS-T_XPR2_, LEU2 | This study |
| PYLβA1 | P_hp4d_-βBS-T_XPR2_, P_hp4d_-A1-T_XPR2_, LEU2 | This study |
| PYLβA2 | P_hp4d_-βBS-T_XPR2_, P_hp4d_-A2-T_XPR2_, LEU2 | This study |
| pYLβHS | P_hp4d_-βBS-T_XPR2_, P_hp4d_-HS-T_XPR2_, LEU2 | This study |
| pYLβHR | P_hp4d_-βBS-T_XPR2_, P_hp4d_-HR-T_XPR2_, LEU2 | This study |
| pYLβMK | P_hp4d_-βBS-T_XPR2_, P_hp4d_-MK-T_XPR2_, LEU2 | This study |
| pYLβPK | P_hp4d_-βBS-T_XPR2_, P_hp4d_-PK-T_XPR2_, LEU2 | This study |
| pYLβPD | P_hp4d_-βBS-T_XPR2_, P_hp4d_-PD-T_XPR2_, LEU2 | This study |
| pYLβIDI | P_hp4d_-βBS-T_XPR2_, P_hp4d_-IDI-T_XPR2_, LEU2 | This study |
| pYLβGS | P_hp4d_-βBS-T_XPR2_, P_hp4d_-GS-T_XPR2_, LEU2 | This study |
| pYLβFS | P_hp4d_-βBS-T_XPR2_, P_hp4d_-FS-T_XPR2_, LEU2 | This study |
| pYLγA1 | P_hp4d_-γBS-T_XPR2_, P_hp4d_-A1-T_XPR2_, LEU2 | This study |
| PYLγA2 | P_hp4d_-γBS-T_XPR2_, P_hp4d_-A2-T_XPR2_, LEU2 | This study |
| pYLγHS | P_hp4d_-γBS-T_XPR2_, P_hp4d_-HS-T_XPR2_, LEU2 | This study |
| pYLγHR | P_hp4d_-γBS-T_XPR2_, P_hp4d_-HR-T_XPR2_, LEU2 | This study |
| pYLγMK | P_hp4d_-γBS-T_XPR2_, P_hp4d_-MK-T_XPR2_, LEU2 | This study |
| pYLγPK | P_hp4d_-γBS-T_XPR2_, P_hp4d_-PK-T_XPR2_, LEU2 | This study |
| pYLγPD | P_hp4d_-γBS-T_XPR2_, P_hp4d_-PD-T_XPR2_, LEU2 | This study |
| pYLγIDI | P_hp4d_-γBS-T_XPR2_, P_hp4d_-IDI-T_XPR2_, LEU2 | This study |
| pYLγGS | P_hp4d_-γBS-T_XPR2_, P_hp4d_-GS-T_XPR2_, LEU2 | This study |
| pYLγFS | P_hp4d_-γBS-T_XPR2_, P_hp4d_-FS-T_XPR2_, LEU2 | This study |
| pYLαHR-Acrb | P_hp4d_-αBS-T_XPR2_, P_hp4d_-HR-T_XPR2_, P_hp4d_-Acrb-T_XPR2_,LEU2 | This study |
| pYLαHR-CMQ | P_hp4d_-αBS-T_XPR2_, P_hp4d_-HR-T_XPR2_, P_hp4d_-CMQ-T_XPR2_,LEU2 | This study |
| pYLβHR-Acrb | P_hp4d_-βBS-T_XPR2_, P_hp4d_-HR-T_XPR2_, P_hp4d_-Acrb-T_XPR2_,LEU2 | This study |
| pYLβHR-CMQ | P_hp4d_-βBS-T_XPR2_, P_hp4d_-HR-T_XPR2_, P_hp4d_-CMQ-T_XPR2_,LEU2 | This study |
| pYLγHR-Acrb | P_hp4d_-γBS-T_XPR2_, P_hp4d_-HR-T_XPR2_, P_hp4d_-Acrb-T_XPR2_,LEU2 | This study |
| pYLγHR-CMQ | P_hp4d_-γBS-T_XPR2_, P_hp4d_-HR-T_XPR2_, P_hp4d_-CMQ-T_XPR2_,LEU2 | This study |

**Table S3** **Strains used in this study**

| Strains | Genotype | Reference |
| --- | --- | --- |
| Po1g KU70Δ | MatA, leu2-270, ura3-302::URA3, xpr2-332, axp-2, ku70- | (Yu *et al.,* 2016) |
| Po1g KαBS | MatA, leu2-270, ura3-302::URA3, xpr2-332, axp-2, ku70-,αBS | This study |
| Po1g KβBS | MatA, leu2-270, ura3-302::URA3, xpr2-332, axp-2, ku70-, βBS | This study |
| Po1g KγBS | MatA, leu2-270, ura3-302::URA3, xpr2-332, axp-2, ku70-, γBS | This study |
| Po1g KαA1 | MatA, leu2-270, ura3-302::URA3, xpr2-332, axp-2, ku70-,αBS, ACOAAT1 | This study |
| Po1g KαA2 | MatA, leu2-270, ura3-302::URA3, xpr2-332, axp-2, ku70-,αBS, ACOAAT2 | This study |
| Po1g KαHS | MatA, leu2-270, ura3-302::URA3, xpr2-332, axp-2, ku70-,αBS, HMGS | This study |
| Po1g KαHR | MatA, leu2-270, ura3-302::URA3, xpr2-332, axp-2, ku70-,αBS, HMGR | This study |
| Po1g KαMK | MatA, leu2-270, ura3-302::URA3, xpr2-332, axp-2, ku70-,αBS, MK | This study |
| Po1g KαPK | MatA, leu2-270, ura3-302::URA3, xpr2-332, axp-2, ku70-,αBS, PMK | This study |
| Po1g KαPD | MatA, leu2-270, ura3-302::URA3, xpr2-332, axp-2, ku70-,αBS, PMVADO | This study |
| Po1g KαIDI | MatA, leu2-270, ura3-302::URA3, xpr2-332, axp-2, ku70-,αBS, IPPDI | This study |
| Po1g KαGS | MatA, leu2-270, ura3-302::URA3, xpr2-332, axp-2, ku70-,αBS, GGPPS | This study |
| Po1g KαFS | MatA, leu2-270, ura3-302::URA3, xpr2-332, axp-2, ku70-,αBS, FPPS | This study |
| Po1g KβA1 | MatA, leu2-270, ura3-302::URA3, xpr2-332, axp-2, ku70-, βBS, ACOAAT1 | This study |
| Po1g KβA2 | MatA, leu2-270, ura3-302::URA3, xpr2-332, axp-2, ku70-, βBS, ACOAAT2 | This study |
| Po1g KβHS | MatA, leu2-270, ura3-302::URA3, xpr2-332, axp-2, ku70-, βBS, HMGS | This study |
| Po1g KβHR | MatA, leu2-270, ura3-302::URA3, xpr2-332, axp-2, ku70-, βBS, HMGR | This study |
| Po1g KβMK | MatA, leu2-270, ura3-302::URA3, xpr2-332, axp-2, ku70-, βBS, MK | This study |
| Po1g KβPK | MatA, leu2-270, ura3-302::URA3, xpr2-332, axp-2, ku70-, βBS, PMK | This study |
| Po1g KβPD | MatA, leu2-270, ura3-302::URA3, xpr2-332, axp-2, ku70-, βBS, PMVADO | This study |
| Po1g KβIDI | MatA, leu2-270, ura3-302::URA3, xpr2-332, axp-2, ku70-, βBS, IPPDI | This study |
| Po1g KβGS | MatA, leu2-270, ura3-302::URA3, xpr2-332, axp-2, ku70-, βBS, GGPPS | This study |
| Po1g KβFS | MatA, leu2-270, ura3-302::URA3, xpr2-332, axp-2, ku70-, βBS, FPPS | This study |
| Po1g KγA1 | MatA, leu2-270, ura3-302::URA3, xpr2-332, axp-2, ku70-,αBS, ACOAAT1 | This study |
| Po1g KγA2 | MatA, leu2-270, ura3-302::URA3, xpr2-332, axp-2, ku70-,αBS, ACOAAT2 | This study |
| Po1g KγHS | MatA, leu2-270, ura3-302::URA3, xpr2-332, axp-2, ku70-,αBS, HMGS | This study |
| Po1g KγHR | MatA, leu2-270, ura3-302::URA3, xpr2-332, axp-2, ku70-,αBS, HMGR | This study |
| Po1g KγMK | MatA, leu2-270, ura3-302::URA3, xpr2-332, axp-2, ku70-,αBS, MK | This study |
| Po1g KγPK | MatA, leu2-270, ura3-302::URA3, xpr2-332, axp-2, ku70-,αBS, PMK | This study |
| Po1g KγPD | MatA, leu2-270, ura3-302::URA3, xpr2-332, axp-2, ku70-,αBS, PMVADO | This study |
| Po1g KγIDI | MatA, leu2-270, ura3-302::URA3, xpr2-332, axp-2, ku70-,αBS, IPPDI | This study |
| Po1g KγGS | MatA, leu2-270, ura3-302::URA3, xpr2-332, axp-2, ku70-,αBS, GGPPS | This study |
| Po1g KγFS | MatA, leu2-270, ura3-302::URA3, xpr2-332, axp-2, ku70-,αBS, FPPS | This study |
| Po1g KαHR-Acrb | MatA, leu2-270, ura3-302::URA3, xpr2-332, axp-2, ku70-,αBS, HMGR, Acrb | This study |
| Po1g KαHR-CMQ | MatA, leu2-270, ura3-302::URA3, xpr2-332, axp-2, ku70-,αBS, HMGR, CMQ | This study |
| Po1g KβHR-Acrb | MatA, leu2-270, ura3-302::URA3, xpr2-332, axp-2, ku70-, βBS, HMGR, Acrb | This study |
| Po1g KβHR-CMQ | MatA, leu2-270, ura3-302::URA3, xpr2-332, axp-2, ku70-, βBS, HMGR, CMQ | This study |
| Po1g KγHR-Acrb | MatA, leu2-270, ura3-302::URA3, xpr2-332, axp-2, ku70-,αBS, HMGR, Acrb | This study |
| Po1g KγHR-CMQ | MatA, leu2-270, ura3-302::URA3, xpr2-332, axp-2, ku70-,αBS, HMGR, CMQ | This study |

**Table S4** Primers used in PCR

| Primer | Sequence (5’-3’) |
| --- | --- |
| α-F | ACAACCACACACATCCACAATGGCCGGTGTCTCTGCC |
| α-R | GGGACAGGCCATGGAGGTACCTTAGTGGTGATGGTGGTGGTGG |
| β-F | ACAACCACACACATCCACAATGGAACTCGTTGATACTCCCTC |
| β-R | GGGACAGGCCATGGAGGTACCTTAGTGATGATGATGGTGGTGG |
| γ-F | ACAACCACACACATCCACAATGTCTATCTCCTCTTCCCCCTC |
| γ-R | GGGACAGGCCATGGAGGTACCTTAGTGGTGATGGTGGTGGTGC |
| ACOAAT1-F | ACAACCACACACATCCACAATGCTACTCGACAGAAGAGACCTT |
| ACOAAT1-R | ACAAGTTCCGTAGTTGGATCCCTAATGCGACTCACTCTGCCCC |
| ACOAAT2-F | ACAACCACACACATCCACAATGGAGCCCGTCTACATTGTTTC |
| ACOAAT2-R | GGGACAGGCCATGGAGGTACCCTAACACTTCTCAACAATGATAGAGG |
| HMGS-F | ACAACCACACACATCCACAATGTCGCAACCCCAGAACG |
| HMGS-R | GGGACAGGCCATGGAGGTACCCTACTGCTTGATCTCGTACTTTCGTC |
| HMGR-F | ACAACCACACACATCCACAATGCTACAAGCAGCTATTGGAAA |
| HMGR-R | GGGACAGGCCATGGAGGTACCCTATGACCGTATGCAAATATTCGAA |
| MK-F | ACAACCACACACATCCACAATGGACTACATCATTTCGGCG |
| MK-R | GGGACAGGCCATGGAGGTACCCTAATGGGTCCAGGGACCGA |
| PMK-F | ACAACCACACACATCCACAATGCTACTTGAACCCCTTCTCG |
| PMK-R | GGGACAGGCCATGGAGGTACCCTAATGACCACCTATTCGGCTCC |
| PMVADO-F | ACAACCACACACATCCACAATGATCCACCAGGCCTCCA |
| PMVADO-R | GGGACAGGCCATGGAGGTACCCTACTTGCTGTTCTTCAGAGAACCA |
| IPPDI-F | ACAACCACACACATCCACAATGCTACTTGATCCACCGCC |
| IPPDI-R | GGGACAGGCCATGGAGGTACCCTAATGACGACGTCTTACAGCGA |
| GGPPS-F | ACAACCACACACATCCACAATGTCACTGCGCATCCTCAA |
| GGPPS-R | GGGACAGGCCATGGAGGTACCCTAATGGATTATAACAGCGCGGA |
| FPPS-F | ACAACCACACACATCCACAATGCTACTTCTGTCGCTTGTAAATC |
| FPPS-R | ACAAGTTCCGTAGTTGGATCCCTAATGTCCAAGGCGAAATTCGA |
| BDH2-F | CCATCCAGCCTCGCGTCGGTTAACTATCCTAGGAGGCCGTTGAGCAC |
| BDH2-R | ACGTCTTGCTGGCGTTCGATAAGCTGTCAAACATGAGAATTCG |
| Acrb-BDH-F | ATCCAGCCTCGCGTCGGTTCAAGGAATGGTGCATG |
| Acrb-BDH-R | CGGCCTCCTAGGATAGTTGTTTGACAGCTTATCATCG |
| CMQ-BDH-F | ATCCAGCCTCGCGTCGGTTCAAGGAATGGTGCATGCTGAG |
| CMQ-BDH-R | CGGCCTCCTAGGATAGTTCATCGATGATAAGCTGTCAAAC |
| pYL-F | CCTCGATCCGGCATGCACTGATCACG |
| pYL-R | TAGGCAACAGCGTTGGGAGAGCCCTTGAGG |
| DJY-F | AATCGCCGTGACGATCAGC |
| DJY-R | CTGTCGCTTGCGGTATTCGG |
| YZ-F | AATCGCCGTGACGATCAGC |
| YZ-R | TCGGACATGGTGATCATGC |

**Supplementary Methods**

**Preparation of *Y. lipolytica* Po1g** ***KU70*△ competent cell**

1. Inoculate a colony of *Y. lipolytica* Po1g *KU70*△ strain from a fresh YPD plate in 10 mL YPD medium (1% yeast extract, 2% peptone, 2% dextrose and 50 mM citrate buffer pH 4.0) in a 250 mL flask. Incubate with shaking at 225 rpm and 30°C for 20 hours.

2. Pellet the cells by centrifuging 5 minutes at 5, 000 g at 4℃.

3. Wash the cells with 20 mL TE buffer and pellet the cells similarly as Step 2.

4. Resuspend the cells in 1 mL of 0.1 M lithium acetate (pH 6.0, adjusted with acetic acid) and incubate for 10 minutes at room temperature.

5. Aliquot the competent cells (100 µL) into sterile 1.5 mL tubes. Proceed to the transformation steps below immediately, or add glycerol to a final concentration of 25% (v/v) and store at -80°C for long-term storage.

**Transformation of *Y. lipolytica* Po1g *KU70*△ cells**

1. Gently mix 10 µL of denatured salmon sperm DNA (10 mg/mL) and 1-5 µg of the linearized plasmid together with 100 µL of competent cells, and incubate at 30°C for 15 minutes.

2. Add 700 µL of 40% PEG-4000 (dissolved in 0.1 M lithium acetate pH 6.0), mix well and incubate at 30°C for 60 minutes with shaking (225 rpm).

3. Heat shock the transformation mixture at 39°C for 60 minutes.

4. Add 1 mL YPD medium and recover for 2 hours at 30°C and 225 rpm.

5. Centrifuge at 10,000 g for 1 minute, remove the supernatant and resuspend the pellet in 1 mL of TE buffer.

6. Repellet the cells and discard the supernatant again.

7. Resuspend the pellet in 100 µL of TE buffer and plate onto selective plates (leucine-deficient plates).

**Supplementary Figures**


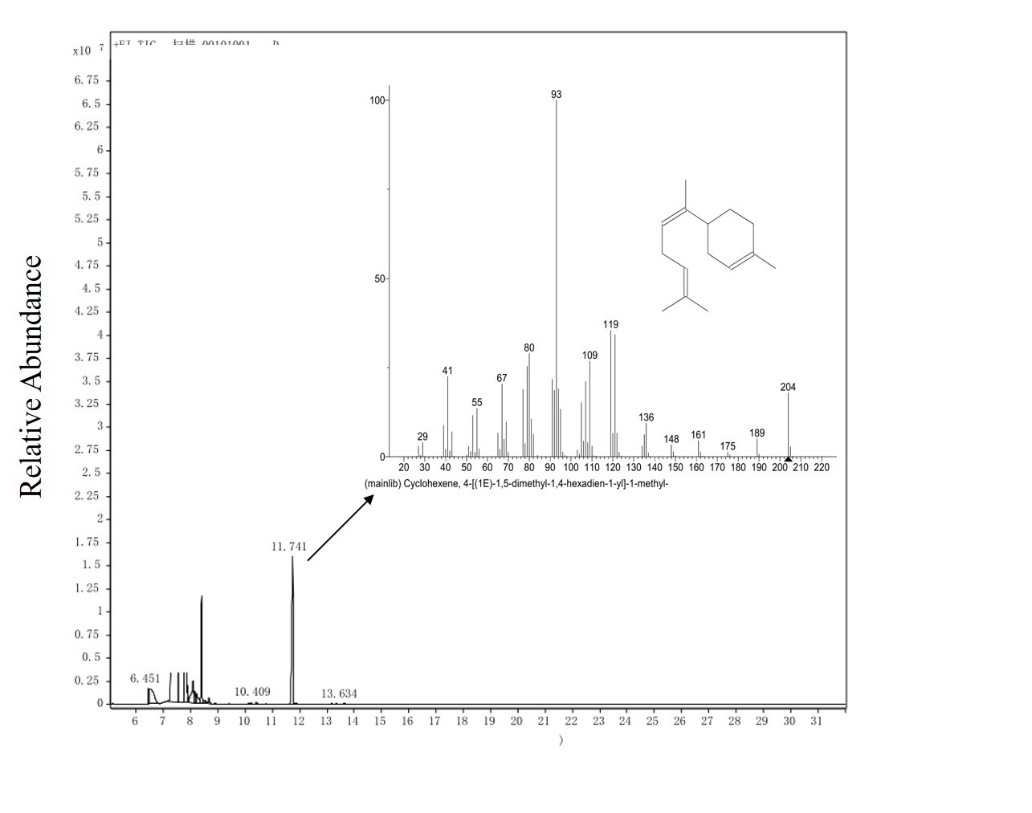
A


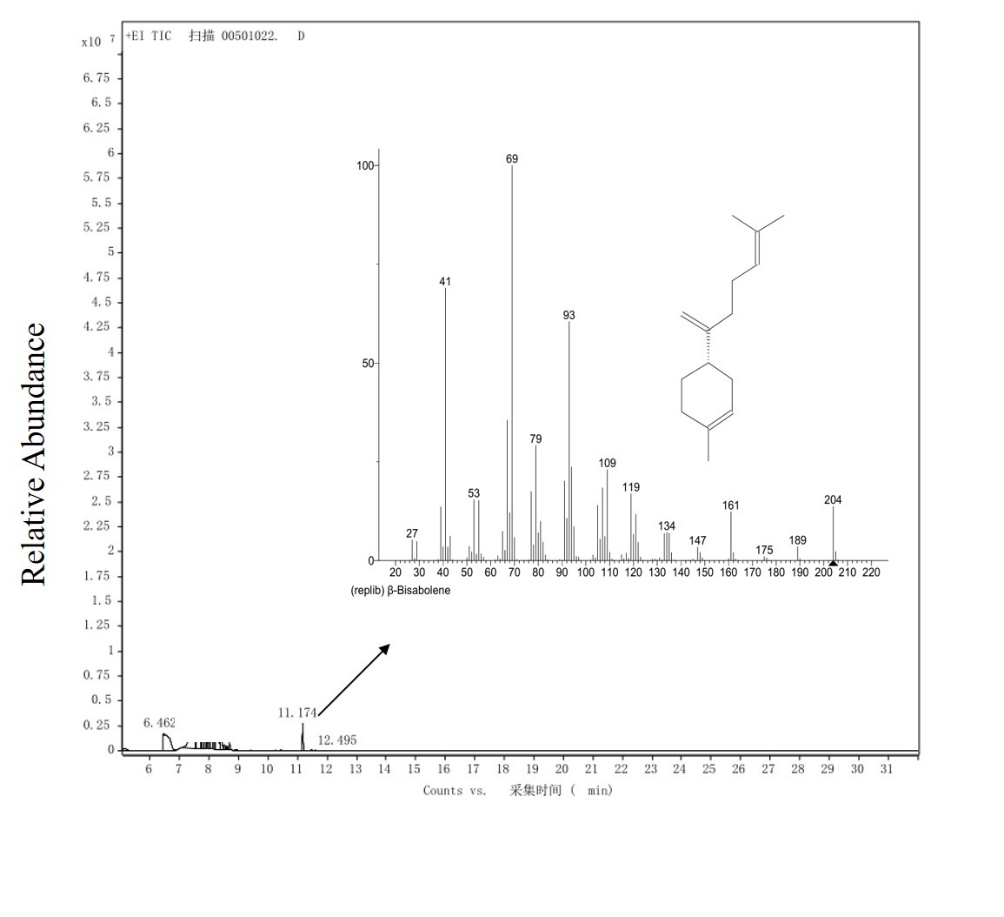
B
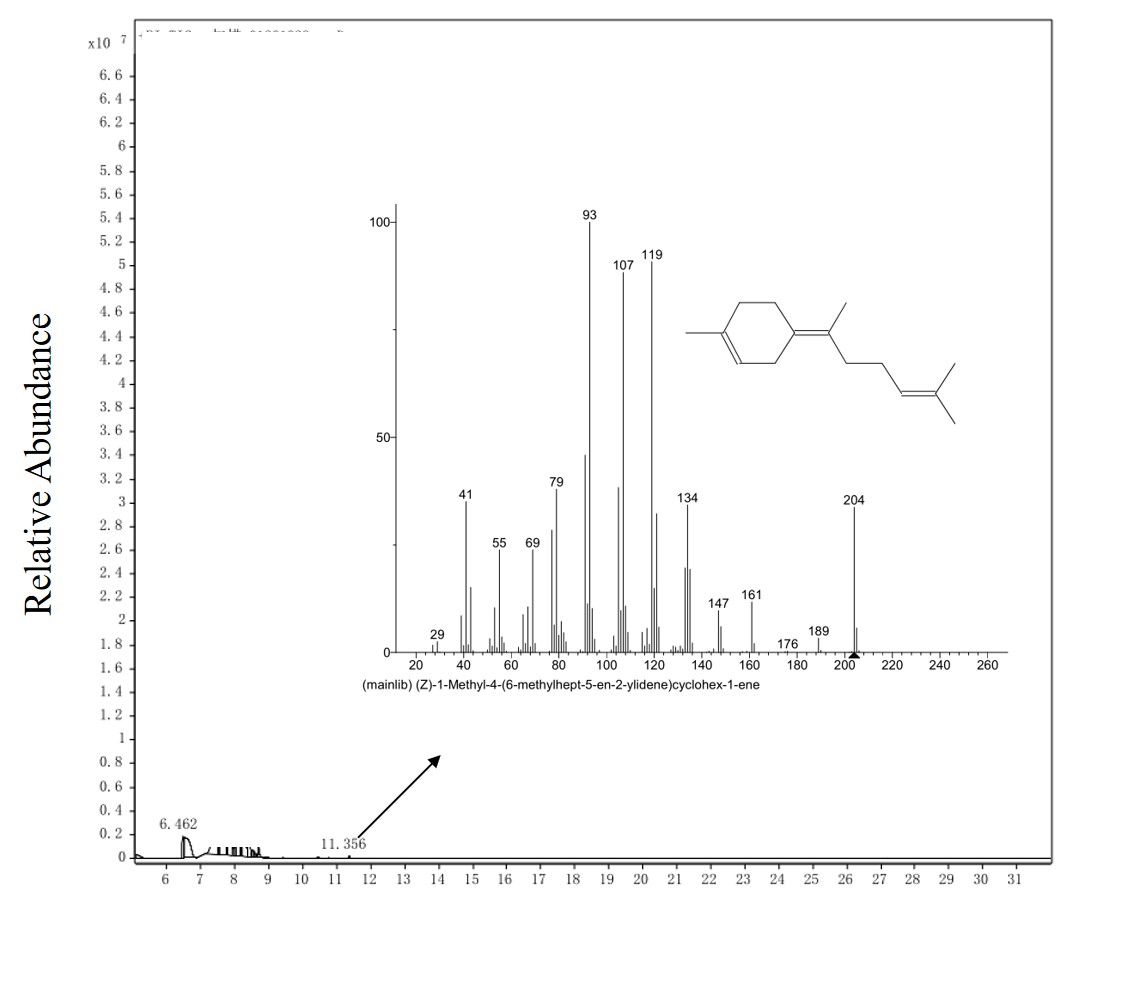
C

**Figure S1.** GC-MS profile of solvent overlay-extracted bisabolenes from cultures of engineered *Y. lipolytica* strains. (A) α-bisabolene obtained in YPD medium of Po1g KαBS1; (B) β-bisabolene obtained in YPD medium of KβBS; (C) γ-bisabolene obtained in YPD medium of KγBS.


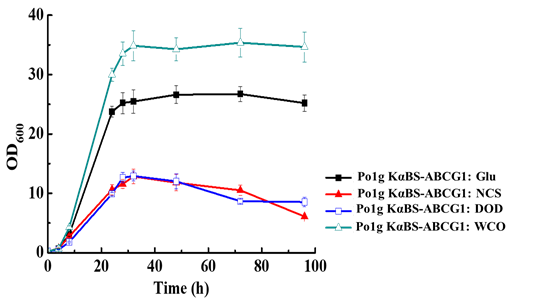


**Figure S2.** Effect of different carbon sources on growth of *Y. lipolytica* Po1g KαBS-ABCG1. Different carbon sources (glucose, waste cooking oil and *n*-dodecane) were added into the base medium, which include 1% yeast extract and 2% tryptone. OD_600_ was measured at 4 h, 8 h, 24 h, 28 h, 32 h, 48 h, 72 h and 96 h. Glu: 2% glucose was added into the base medium; NCS: there was no addition of any external carbon sources in the base medium; DOD: *n*-dodecane was added into the base medium; WCO: 1.18% (w/v) waste cooking oil as a carbon source which has an equal amount of carbon units to that of 2% (w/v) glucose was added into the base medium. All values presented are the mean of three biological replicates ± standard deviation.


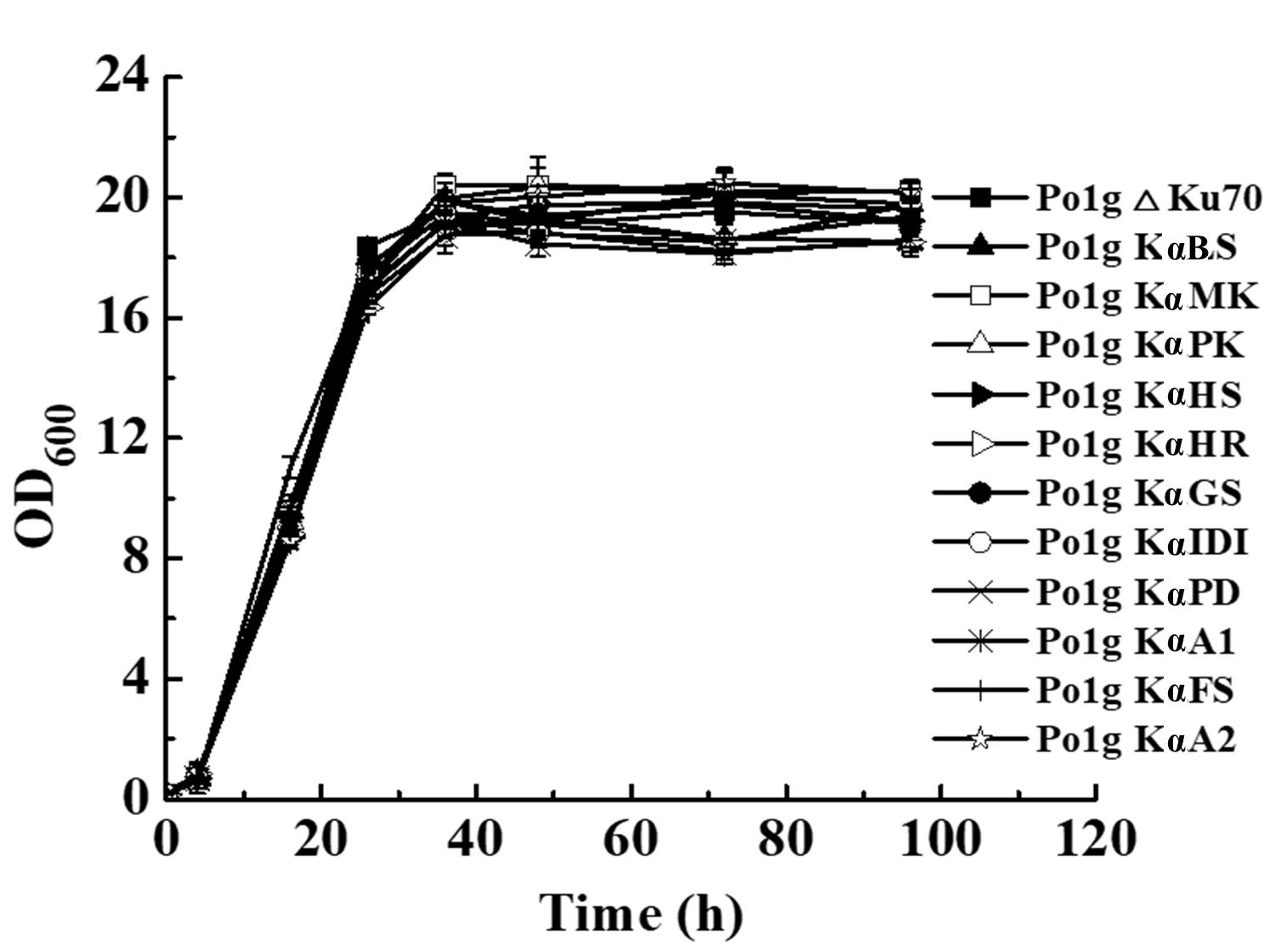


**A**

**
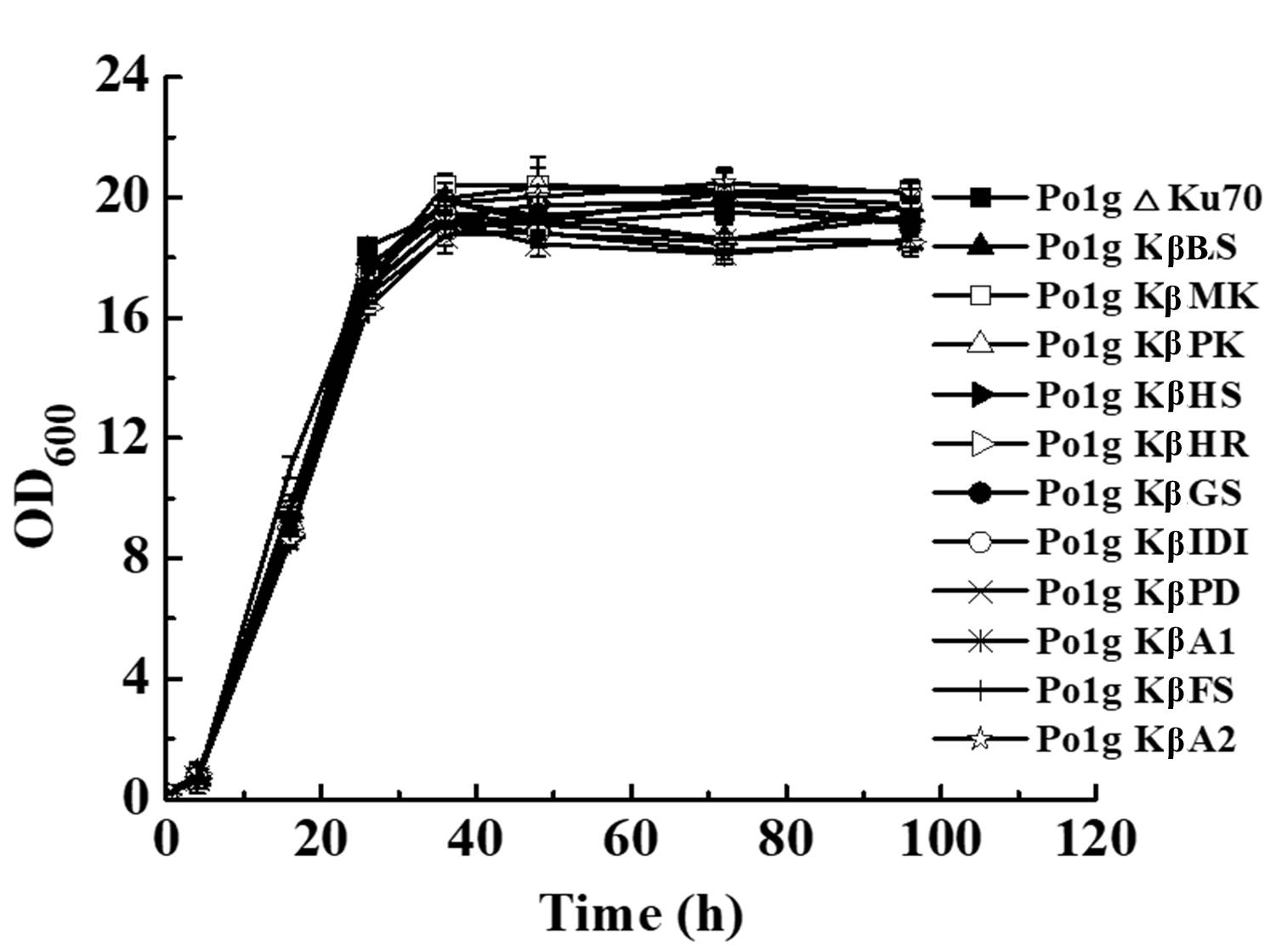
**

**B**

**
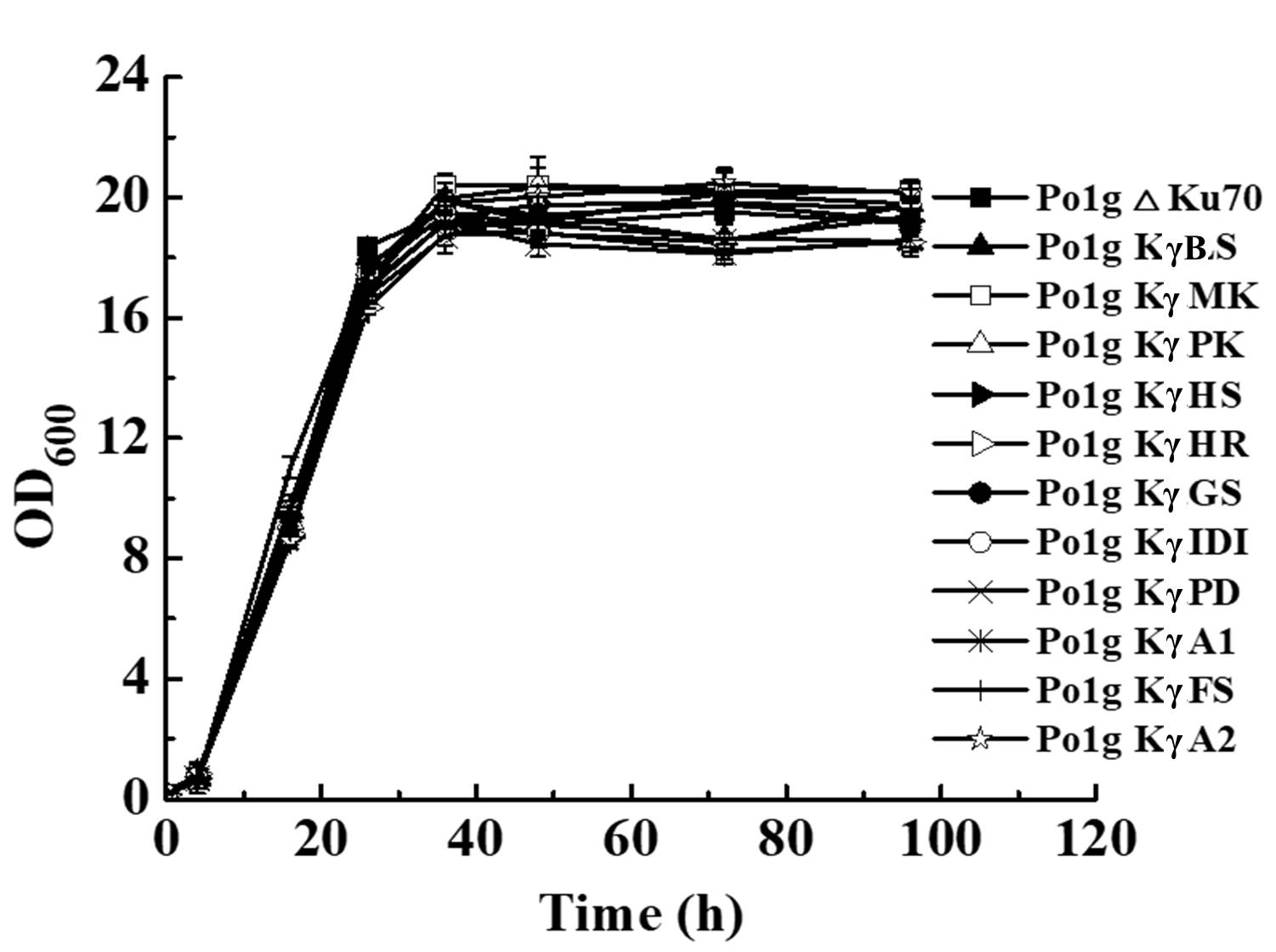
**

**C**

**Figure S3.** The OD_600_ values of 30 engineered *Y. lipolytica* strains cultured in YPD medium (A: α-bisabolene production strains; B: β-bisabolene production strains; γ- bisabolene production strains). OD_600_ were measured at 0, 4, 16, 26, 32, 48, 72, 96 and 120 h, respectively. The cultivation was performed at 28℃ and 220 rpm. All values presented are the mean of three biological replicates ± standard deviation.


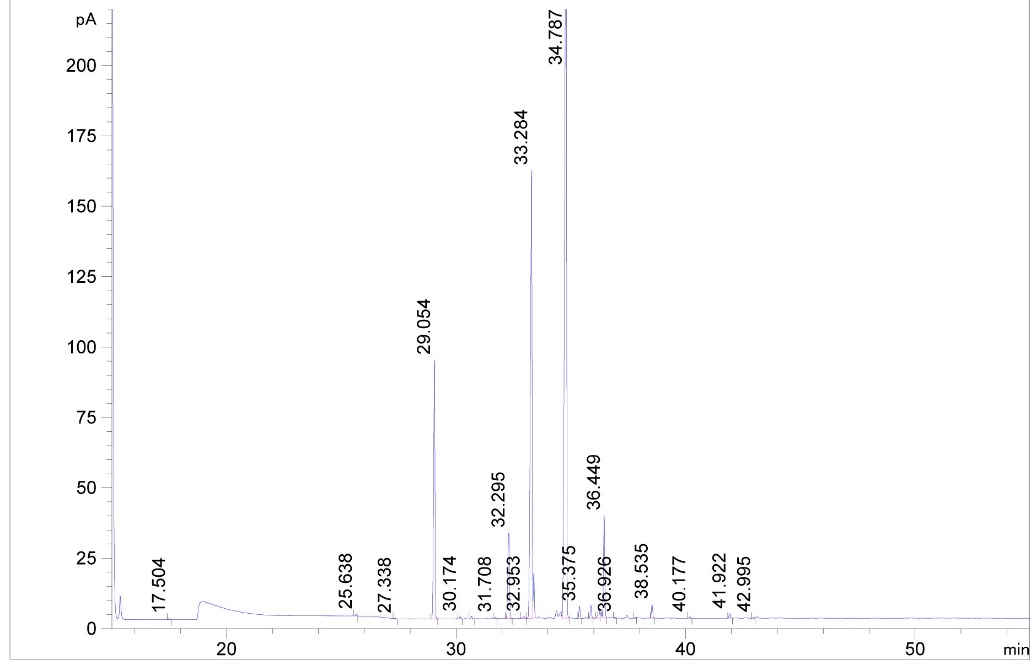


**Figure S4.** The GC-MS analysis of fatty acid composition in waste cooking oil.


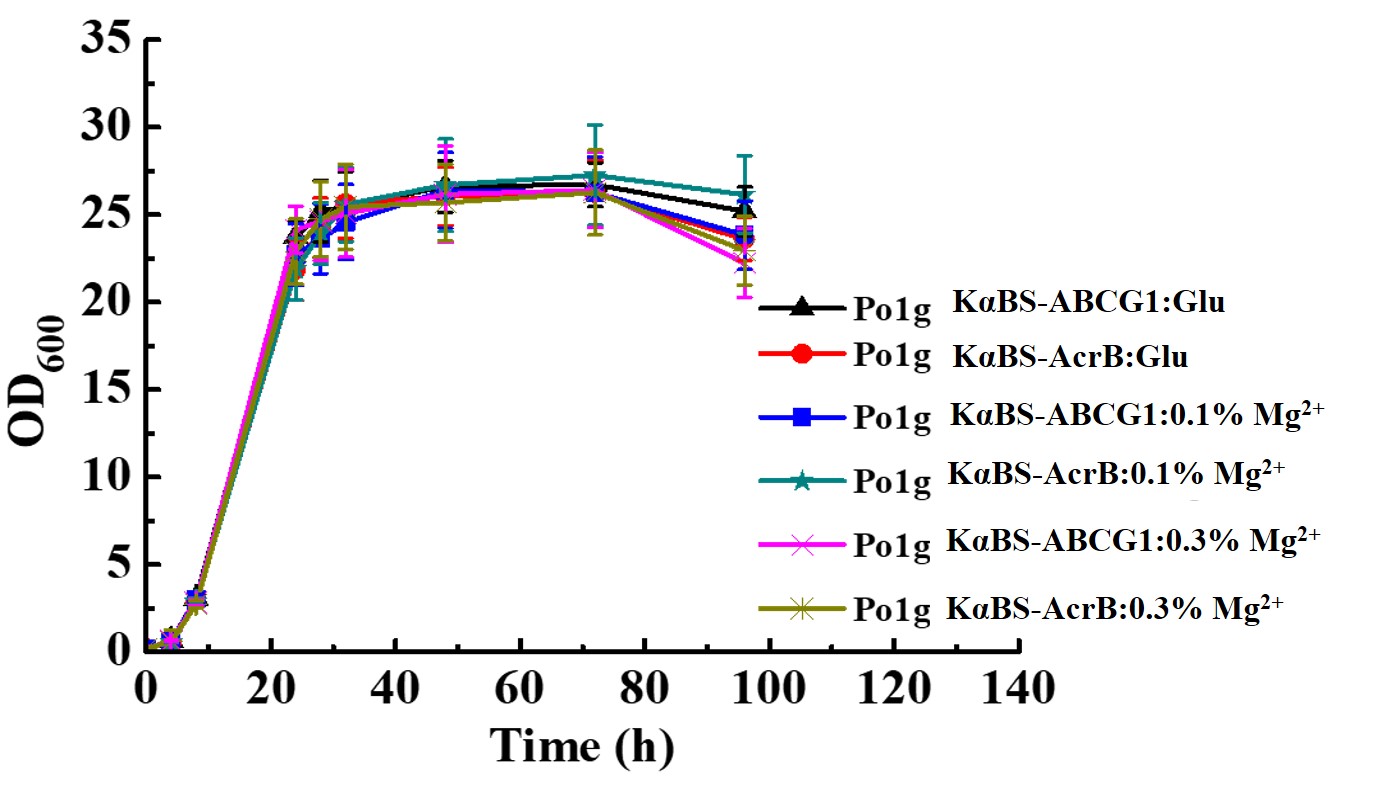


**Figure S5.** Effect of Mg^2+^ on growth of *Y. lipolytica*. Different concentration of MgSO_4_•7H_2_O was added into the medium. OD_600_ was measured at 4 h, 8 h, 24 h, 28 h, 32 h, 48 h, 72 h and 96 h. All values presented are the mean of three biological replicates ± standard deviation.


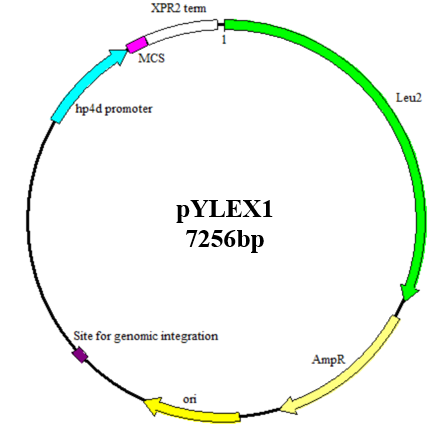


**Figure S6** Map of the plasmid pYLEX1.
